# Preparation for online psychological therapy for depression in people living with and beyond cancer in East Midlands NHS primary and secondary care services in England: protocol for the PROSPER randomised controlled trial

**DOI:** 10.1136/bmjopen-2025-108442

**Published:** 2026-05-18

**Authors:** Clement Boutry, Paulina Hagyari-Donaldson, Adam Hill, Fallon Mauger, Chloe Mays, Catherine Macauley, Melissa Covington, Rebecca Wynn, Karen Simpson, Melanie Cordrey, Fred Higton, Charlotte L Hall, Nima Moghaddam, Richard Morriss, James Rathbone, Boliang Guo, Shireen Patel, Sam Malins

**Affiliations:** 1 Institute of Mental Health, School of Medicine, University of Nottingham, Nottingham, England, UK; 2 Nottinghamshire Healthcare NHS Foundation Trust, Nottingham, UK; 3 Psychological Health and Wellbeing (PHeW) Group, University of Lincoln, Lincoln, England, UK

**Keywords:** ONCOLOGY, Depression & mood disorders, Randomized Controlled Trial

## Abstract

**Introduction:**

Depression is up to four times more common among individuals with cancer compared with the general population. Psychological therapies are effective in treating depression among patients in cancer care, but access is often delayed, which can exacerbate symptoms, increase dropout and reduce therapeutic effectiveness. This study evaluates the clinical and cost-effectiveness of a therapy preparation intervention (TPI) designed to enhance engagement and outcomes among patients awaiting psychological therapy in cancer care.

**Methods and analysis:**

This study is a parallel-group, two-arm, multicentre, single-blind randomised controlled trial. A total of 150 adults (≥18 years) living with or beyond cancer and experiencing moderate-to-severe depression will be recruited from health services in the East Midlands region of England. Participants will be randomised (1:1) to receive either TPI plus treatment as usual (TAU) or TAU alone.

The primary outcome is depression severity measured using the Patient Health Questionnaire 9-items (PHQ-9) over a 24-week follow-up. Secondary outcomes include anxiety, functioning, mental well-being, patient activation, readiness for change, health-related quality of life and health economics, and therapy engagement including attendance and dropout. Hope and in-session patient activation, assessed using recorded treatment preparation sessions, will be explored as additional mechanistic variables. Health economic outcomes will be assessed at baseline and 24 weeks. Data will be collected via online or telephone surveys at baseline, and at 4, 8, 12 and 24 weeks post randomisation. Qualitative interviews with a subset of participants will explore intervention experiences, analysed using reflexive thematic analysis.

**Ethics and dissemination:**

Ethical approval has been obtained from the Health Research Authority and National Health Service Research Ethics Committee (Bromley) (REC reference: 24/LO/0610). Findings will be disseminated through peer-reviewed journals, academic conferences and clinical and patient networks.

**Trial registration number:**

ISRCTN registry: ISRCTN13692666, registered on 18 October 2024.

**STRENGTHS AND LIMITATIONS OF THIS STUDY**

- The pragmatic randomised controlled trial design evaluates the intervention under real-world conditions, enhancing generalisability and clinical relevance.
- Integration of clinical outcomes and mechanistic assessments using a mixed methods design can provide a more comprehensive understanding of outcomes and underlying mechanisms.
- Embedding the intervention within existing UK National Health Service pathways supports scalability and potential future implementation. Remote delivery improves accessibility and cost-effectiveness but may pose challenges for participants with limited digital literacy or access.
- Reliance on self-reported measures may introduce bias, particularly among participants undergoing intensive cancer treatments. Besides, as the intervention includes personalised messaging, any observed effects may reflect both motivational interviewing (MI)-consistent mechanisms and non-specific attention/contact effects; this will be considered when interpreting findings.
- Detailed oncology characteristics (eg, tumour site, stage and current treatment modality) are not collected as baseline trial variables, which may limit subgroup interpretation and may increase heterogeneity in outcome estimates; nevertheless, these data will be useful for informing future trial design and moderator hypotheses.

## Introduction

### Background and rationale

 The prevalence of common mental health problems among people diagnosed with and treated for cancer is up to four times higher than that observed in the general population,^1^ leading to an elevated risk of suicide and diminished quality of life lasting for more than 10 years after diagnosis.^2 3^ These difficulties often go untreated, which has multiple deleterious effects including negative impacts on the patients’ concordance with cancer treatment.^4 5^ Evidence-based psychological therapies, such as cognitive behavioural therapy (CBT), are shown to be effective in improving mental health outcomes for people living with and beyond cancer.^6^ However, timely access to these interventions is a key challenge. Patients referred for psychological therapies typically wait several weeks or months before gaining access to treatment^7^ and the longer patients wait, the greater the risk of their mental health worsening.^8^,^12^

Patients’ engagement with treatment can also be affected by long waits. For example, only 55% of people accessing UK National Health Service (NHS) Talking Therapies for anxiety and depression attend more than one session.^13^ For people living with and beyond cancer, therapy engagement can be even more challenging due to the multiple competing demands of managing their physical health, treatment side effects and cancer-related stressors.^14^ Moreover, the likelihood of dropout increases when patients experience long wait times without any input,^15^ highlighting the need for interventions that support engagement from the point of referral. Besides dropout rates, there are wider engagement challenges in ensuring that patients fully use therapy to achieve the greatest benefits, such as potential ambivalence about change.^16^

One approach to maximising responsiveness to psychological therapy is the use of brief preparatory interventions, which aim to clarify expectations and empower patients to optimise the benefits of subsequent therapy.^17^ They aim to increase engagement and responsiveness to subsequent psychotherapy by clarifying what therapy involves, aligning expectations and supporting patients to anticipate and problem-solve practical and psychological barriers to engagement such as confidence, ambivalence and competing demands.^16 17^ These interventions are often delivered in a single structured session and may be augmented with brief written or digital materials.^17^ Evidence from wait-list contexts suggests that offering a brief intervention during the waiting period may initiate meaningful change and may mitigate deterioration while patients await treatment.^12 18^

In addition to improving therapy attendance, brief preparatory interventions have been shown to enhance therapy outcomes and initiate meaningful change,^19^,^21^ while those on waiting lists often see little improvement and may experience worsening symptoms while they wait.^22^ Overall, existing evidence supports the effectiveness of such single-session interventions in mental healthcare.^20^ When combined with standard therapy, these interventions may contribute to achieving successful therapy outcomes more quickly, including an enhanced management of and a decrease in depressive symptoms.^21^ However, little evidence exists on this type of intervention applied to psychological support within cancer care.

Motivational interviewing (MI) is a particular way of talking with people about change and growth to strengthen their own motivation and commitment.^23^ It was selected as the core approach for this preparatory intervention because it is specifically designed to address ambivalence and strengthen an individual’s own motivation and commitment to change. These factors are highly relevant when depressive symptoms such as reduced energy, low confidence and hopelessness may reduce readiness to start and engage with therapy.^24^ MI aims to initiate and support lasting behavioural change through mechanisms that include fostering hope, enhancing readiness for change and enabling patient activation (an individual’s perceived confidence and ability to manage their health).^25^ In the context of psychological therapy, brief preparation prior to starting treatment can clarify what therapy involves, align expectations and clarify how to gain the most benefit from therapy, which can reduce dropout and improve outcomes.^26^ Additionally, MI can be used to elicit personally meaningful goals for therapy, increase confidence in ability to engage, and proactively identify and problem-solve barriers likely to arise during a waiting period, thereby supporting subsequent uptake and engagement.^16 17^ This is known as role induction and can be delivered as part of a single-session pre-therapy intervention.

MI-based preparation has demonstrated benefits for engagement and/or outcomes when used as a pretreatment integrated with CBT, including trials in anxiety disorders.^19 27^ Existing research also shows the effectiveness of MI for brief or single-session delivery in behaviour change contexts.^28^,^30^ In cancer-related contexts, MI has been used to reduce dropout from psychological interventions delivered to cancer and chronic pain populations.^31^ However, evidence evaluating MI-based preparation delivered specifically during waiting periods for psychological therapy within cancer pathways remains limited, supporting the need for further research.

The effectiveness of therapy may be further improved by combining adjunctive single-session interventions with technology. Current evidence suggests that integrating technology such as smart text messaging with single sessions could improve outcomes and adherence.^32 33^ In cancer care specifically, integrated smart messaging may support psychological therapy engagement, responsiveness and retention of benefits.^34 35^ However, no current evidence exists on the use of integrated technology with single-session interventions in cancer care for those awaiting psychological therapy.

Considering the common and widespread problems associated with waiting times for psychological therapy access,^36^ including the well-evidenced observation that symptoms tend to deteriorate during the waiting period,^10^ interventions that may mitigate these negative impacts warrant investigation. This trial aims to assess the clinical effectiveness of an intervention combining a single-session intervention with smart text messages, designed to help prepare adult patients living with or beyond cancer diagnosis who are awaiting psychological therapy for depression.

### Hypothesis

We hypothesise that participants receiving the therapy preparation intervention (TPI) plus treatment as usual (TAU) will show greater reductions in depression symptoms, lower dropout from psychological therapy and improved secondary outcomes including anxiety, functioning, mental well-being, patient activation, readiness for change and health-related quality of life compared with those receiving TAU alone. Additionally, we expect TPI participants to demonstrate greater improvement in hope and in session indicators of activation and engagement.

### Objectives

The overall aim is to assess the clinical and cost effectiveness of a TPI in terms of its effects on depression, anxiety, functioning, mental well-being, patient activation, readiness for change, health-related quality of life, health economic outcomes and therapy engagement including dropout, compared with usual care.

#### Primary objective

The primary objective is to evaluate the difference in depression symptom scores across the 24-week follow-up period. This comparison is between two arms: TPI plus the standard psychological treatment within the host service (TAU) and TAU alone.

#### Secondary objectives

1. To evaluate differences between trial arms across the 24-week follow-up period in anxiety, functioning, mental well-being, patient activation, readiness for change and health-related quality of life.
2. To evaluate differences between trial arms in therapy engagement, including attendance and dropout.

#### Mechanism of action

To explore differences between trial arms in hope and in session patient activation and engagement, including coded Treatment Preparation Session content.

## Methods and analysis

### Study design

**Figure 1:**
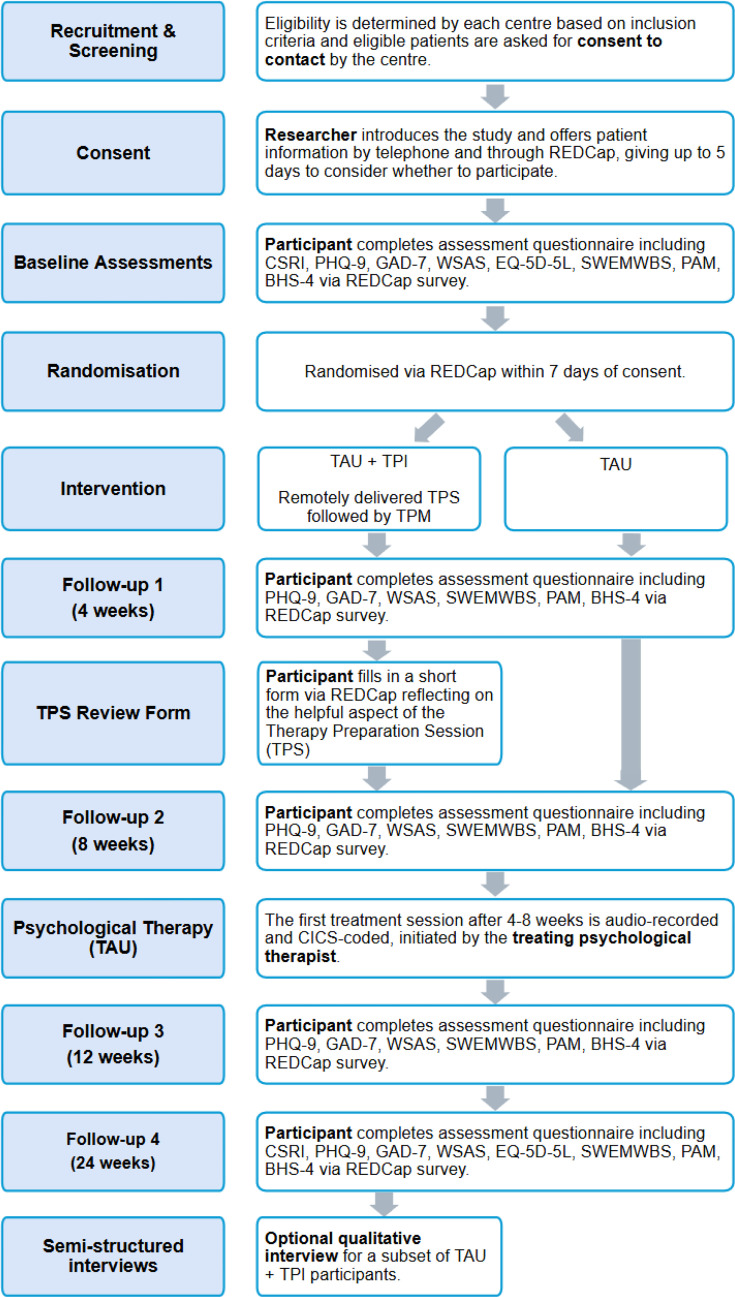
Trial flow chart. Flow and schedule of enrolment, allocation, intervention delivery and outcome assessments for participants in TPI plus TAU versus TAU. BHS-4, Beck’s Hopelessness Scale; CICS, Consultation Interactions Coding Scheme; CSRI, Client Service Receipt Inventory; EQ-5D-5L, EuroQol 5-Dimensions 5-Levels; GAD-7, Generalised Anxiety Disorder 7; PAM, Patient Activation Measure; PHQ-9, Patient Health Questionnaire 9; SWEMWBS, Short Warwick-Edinburgh Mental Wellbeing Scale; TAU, treatment as usual; TPI, therapy preparation intervention; TPM, therapy preparation messaging; TPS, therapy preparation session; WSAS, Work and Social Adjustment Scale.

The PROSPER study is a two-arm, multicentre, parallel-group, single-blind randomised controlled trial comparing TPI+TAU versus TAU alone over 24 weeks for moderate-to-severe depression in current or former cancer care patients in the East Midlands region of England. Participants will be recruited from existing psychological therapy referral pathways across primary and secondary NHS cancer care services, including secondary care cancer centres and primary care general practices. All participants will provide informed consent prior to being randomised. Patient flow and study procedures are shown in figure 1. The study is overseen by an independent Trial Steering Committee (TSC), with the same group acting as a Data Monitoring Committee (DMC). The trial will be reviewed every 6 months through reports to this committee and presentations of progress to the committee by the study team. Participants in both arms will receive the same outcome measures. Follow-ups will occur at 4, 8, 12 and 24 weeks post randomisation. A nested semistructured interview study will explore participants’ experiences with TPI+TAU. Interviews will be conducted no earlier than 2 weeks after the 24-week follow-up assessment is due.

### Participant eligibility and recruitment

Participants will be screened by their clinical care team in primary and secondary care based on the following criteria:

#### Inclusion criteria

- Aged 18 years or older. There is no maximum age limit.
- Able to engage with psychological therapy sessions conducted in English.
- Capable of giving informed consent.
- Have a diagnosis of cancer (past or present): participants may be receiving active anticancer treatment or be in post-treatment surveillance during the waiting period for psychological therapy; treatment status is not used as an eligibility restriction in this pragmatic trial and is not collected as a baseline trial variable.
- Experiencing difficulties with depression in relation to cancer diagnosis, with a score of ≥10 on the Patient Health Questionnaire 9-items (PHQ-9), indicative of at least moderate clinical severity of depressive symptoms.^37^

#### Exclusion criteria

- Immediate risk to self or others.
- Currently receiving psychological therapy with another service.
- Unable or unwilling to receive care remotely.

Participants may be receiving active anticancer treatment or be in post-treatment surveillance during the waiting period for psychological therapy. For the purposes of this trial, ‘cancer’ is defined as a clinician-recorded diagnosis of malignant disease (solid tumour or haematological malignancy), either current or previous (‘living with or beyond cancer’), confirmed in the participant’s clinical record/referral information. Eligibility is not restricted by tumour site, histological subtype, disease stage (including metastatic disease) or treatment status. Participants may be receiving active anticancer treatment (eg, chemotherapy, radiotherapy, immunotherapy, targeted therapy or hormonal therapy) or maintenance treatment or be in post-treatment surveillance at the time of enrolment.

Participants with comorbid mental health conditions such as anxiety are eligible, reflecting routine clinical presentations in NHS psychological therapy pathways. Eligibility decisions are made by the referring clinical team during screening based on clinical appropriateness and safety; individuals requiring urgent/crisis care are excluded via the ‘immediate risk to self or others’ criterion.

A total of 150 participants were planned for recruitment from East Midlands primary and secondary care services. Recruitment commenced in October 2024 and closed in August 2025. Referring clinicians will identify eligible patients and seek written or verbal consent to be contacted by the study researchers who will explain the study, answer questions and email a link to the participant information sheet (PIS) and the informed consent form via the REDCap platform (both the PIS and consent form can be found in online supplementary material X1). Once written informed consent is provided, potential participants will be able to complete the baseline assessment questionnaires on REDCap. Participants who do not have access to the internet will have the option for the PIS and consent form to be posted out to them. Verbal informed consent will be received over the telephone or videoconferencing and will be recorded. The baseline assessment will be conducted over the telephone with a member of the research team and the REDCap questionnaires will be completed during the call. Following completion of the baseline assessments, participants are randomly allocated to either the intervention group (TPI+TAU) or the control group (TAU alone).

### Randomisation and blinding

Participants will be randomly allocated in a 1:1 ratio using a secure web-based randomisation system hosted by the University of Nottingham, UK. The allocation sequence is generated using a Clinical Database Support Service (CDSS) script, with minimisation based on study site and baseline Generalised Anxiety Disorder assessment 7-items (GAD-7) score (clinical cut-off score: ≥8). The CDSS script extracts data from REDCap, applies minimisation and updates allocation while keeping assessors blinded.

Randomisation is expected to occur within seven working days of consent, and participants allocated to the TPI arm will be contacted within 7 days of randomisation to schedule their TPI session. Randomisation is triggered automatically after the baseline assessment, and the randomisation system ensures that those accessing outcome data cannot infer group allocation, as all participants complete identical assessments. Researchers involved in assessment and analysis, including the trial statistician, will remain blinded to allocation throughout the study, while only the trial manager and administrative staff from the host clinical service—East Midlands Cancer Alliance Centre for Psychosocial Health (EMCA CPH)—will have access to allocation to coordinate intervention delivery. The unblinded trial manager will notify the EMCA CPH team of the allocation, who will then schedule the TPI session, confirm arrangements with the participant and coordinate psychological therapy accordingly.

Due to the nature of the intervention, participants and the clinical team are not blinded. Identical assessments are used for all participants, and only the trial manager and administrative staff have access to allocation. Unblinding will only occur at the end of the trial or in the case of a medical emergency or serious adverse event (SAE), with permission from the chief investigator (CI) and carried out by the EMCA CPH administrator. Blinding of outcome assessors will be maintained until data collection is complete.

### Intervention

**Figure 2:**
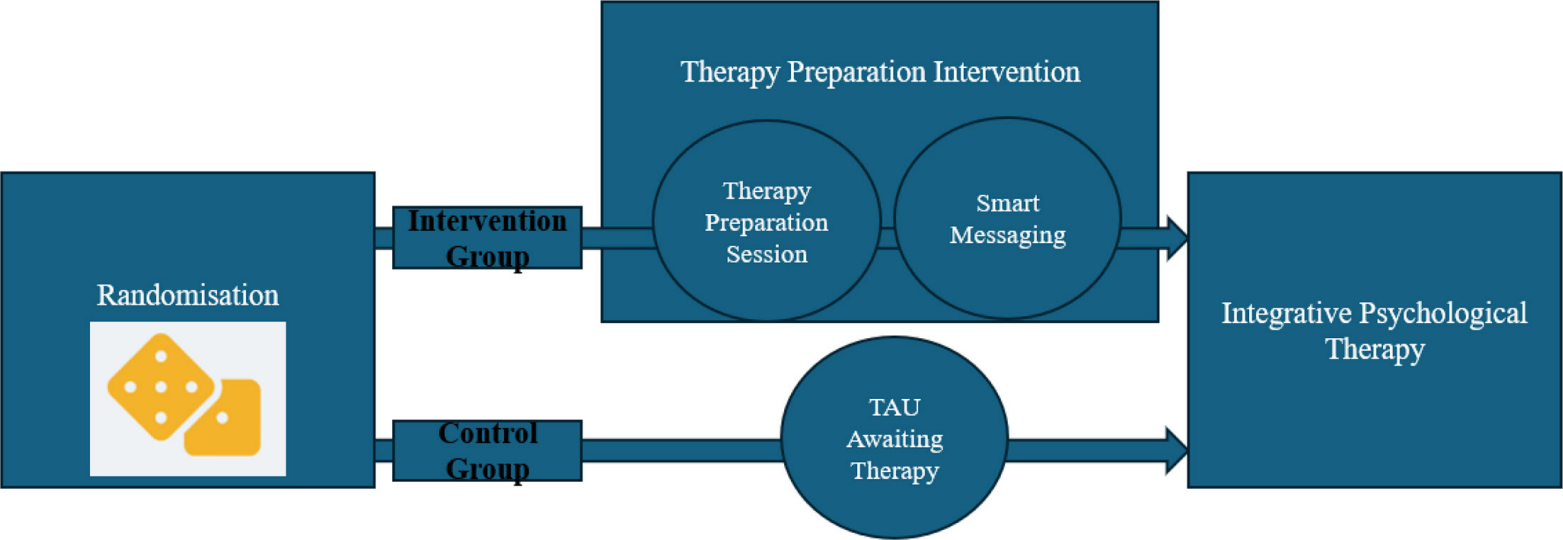
Participant treatment pathway from randomisation in each group. TAU, treatment as usual.

Participants randomised to the intervention arm will receive the TPI in addition to TAU (described in online supplementary material 2). The TPI comprises the therapy preparation session (TPS): a single session delivered remotely (via telephone or video call) for up to 60 min; and the therapy preparation messaging (TPM): 6 weeks of follow-up smart messaging, designed to prepare patients and enhance engagement with therapy during and after the waiting period using personalised reminders from the content of the TPS (see figure 2). The TPS draws on MI, role induction and goal-setting principle. The TPM delivers participants’ individualised engagement strategies and therapy goals identified at the TPM. Full intervention content, delivery procedures and theoretical framework are provided in online supplementary material 3.

Schematic of TPI components and timing, showing the therapy preparation session (TPS) and the therapy preparation messaging (TPM) delivered over 6 weeks alongside usual care.

TAU will involve receiving psychological therapy via video or telephone for depression (with or without anxiety) and includes a typical waiting period of 4–12 weeks from the point of referral. TAU includes routine service contacts and standard written information provided at the start of the waiting period. In both trial arms, outcome data are collected at 4, 8, 12 and 24 weeks post randomisation using identical procedures, including automated REDCap email reminders for questionnaire completion. Therapy is delivered by a practitioner psychologist following an integrative approach, drawing primarily on second-wave and third-wave CBT approaches (with some variability between therapists). On average, the psychological therapy lasts for 8–12 sessions.

At the start of the waiting period for psychological therapy, all participants are provided with a written guide aimed at helping them gain the maximum benefit from their therapy sessions. This document encompasses an overview of the service, expectations for sessions (including attendance and between session tasks), an introduction to confidentiality and privacy concepts, and practical guidelines. These practicalities include session preparation tips such as checking internet connections, being ready for calls in advance (particularly ensuring a confidential and comfortable environment) and guidance on addressing potential technical difficulties.

Adherence to the TPS guidance will be reviewed regularly through examination of TPS recordings in each practitioner’s clinical supervision. Fidelity will be assessed on a sample of TPS recordings for each of the key elements described above.

### Outcomes

The outcomes assessed in this trial comprise primary, secondary, engagement, exploratory mechanistic, qualitative, adherence, safety and economic outcomes.

#### Primary outcomes

The primary outcome is change on the PHQ-9^37^ score across 4-week, 8-week, 12-week and 24-week follow-ups. Follow-up dates will be calculated from the date of randomisation.

#### Secondary outcomes

Secondary outcomes will include changes in the following measures across the same time points (4, 8, 12 and 24 weeks post randomisation):

- Seven-item Generalised Anxiety Disorder Scale (GAD-7).^38^
- Eight-item Work and Social Adjustment Scale.^39^
- Seven-item Short Warwick-Edinburgh Mental Wellbeing Scale.^40^
- Three-item readiness for change ruler.^38^
- Thirteen-item Patient Activation Measure.^41^

#### Economic outcomes

In addition, health-related quality of life will be assessed at 24 weeks using the Visual Analogue Scale of the EuroQol 5-Dimensions 5-Levels (EQ-5D-5L).^42^ Health utility indices will be derived from the EQ-5D-5L for cost-effectiveness analyses (see online supplementary material 4).

#### Engagement outcomes

Engagement will be assessed via therapy attendance records and dropout rates, comparing TPI plus TAU with TAU alone.

#### Mechanisms of action

Differences in psychological mechanisms between trial arms will be assessed using scores collected at 4-week, 8-week, 12-week and 24-week follow-ups for the following outcome measures:

- Four-item Beck Hopelessness Scale.^43^
- The TPS will be recorded and the Consultation Interactions Coding Scheme, an instrument designed to rate psychological therapy interactions in terms of in-session patient activation,^35^ will be used to explore and categorise the content of the sessions.

#### Qualitative outcomes

A subset of participants randomised to TPI+TAU will be invited to take part in an optional 30-minute telephone or videocall interview to explore their experiences of the TPI.

Online supplementary material 5 outlines the completion time points for each measure.

### Adherence measures

Adherence is defined as the extent to which participants engage with the prescribed components of the TPI, including attendance at the TPS, and ongoing participation in psychological therapy. This will be assessed through dropout rates and TPS and session attendance over the course of therapy.

As is normal practice within the host clinical service, participants will be asked to refrain from receiving any talking therapy outside of that provided within the trial for the duration of their participation. This adherence will be verified during the screening phase and reinforced during the agreement stage of their therapy with the host service.

### Safety outcome measures

Data on the impact of the intervention on reducing depression and other outcome measures will not be analysed until the end of the study period and therefore will not inform decisions to stop the research. If participant responses or communications raise concern about safety (eg, deterioration or risk), the research team will follow predefined escalation procedures, including liaison with the participant’s clinical team and signposting to urgent support in line with NHS care pathways.

Adverse events (AEs) are any medical or psychological events experienced by a participant during a study, regardless of whether they are caused by or related to the intervention being tested, and requiring documentation and independent oversight to ensure participant safety, while SAEs have significant outcomes such as death or hospitalisation. Participants may report AEs directly to researchers, during assessments, or via clinicians. Any AEs will be assessed for seriousness and causality by the CI, recorded and monitored until resolution. SAEs will be reported to the CI and study sponsor within 24 hours, and their relevance to the intervention will be determined with clinical input. Participants may be withdrawn at the CI’s discretion if necessary.

All SAEs will be reviewed by the CI and study sponsor and, if there is any indication that these are linked to the intervention, consideration will be given to stopping on the advice of the TSC, the National Institute for Health Research Applied Research Collaboration East Midlands (NIHR ARC-EM) Scientific Committee and study sponsor. For quality assurance, the sponsor, ethics committee or an independent study monitor may audit trial conduct through site visits. During such visits, direct access to source data and trial documentation will be granted, with all parties maintaining strict confidentiality of participant data.

### Data collection and management

Data will be collected and managed using REDCap. Each participant will be assigned a unique trial identification number to maintain confidentiality; identifying information will be stored separately from outcome data. Online assessments will be completed by participants, while phone assessments will be entered by researchers. Only researchers in contact with participants and the EMCA CPH administrative team will access identifiable data. All data will be stored securely and handled in compliance with data protection regulations, sponsor policies and ethical standards.

Participants will complete a baseline assessment and follow-ups at weeks 4, 8, 12 and 24 post randomisation. Surveys should be completed within 7 days of the due date. REDCap will send automated emails 1 week prior, with reminders 4 days after the due date if incomplete. Assessments not completed within 7 days will be marked as missed but not considered a withdrawal unless formally notified. Participants missing the final assessment without contact will be classified as lost to follow-up.

### Statistical methods

More information about the statistical methods can be found in the statistical analysis plan (SAP), in online supplementary material 6.

#### Sample size calculation

Based on the established Minimally Clinically Important Difference on the PHQ-9 (~3 points or ~20% of baseline severity^39^) and consistent with previous trials in cancer patients,^40^ 90 participants are needed to detect a 3-point difference with 90% power at α=0.05. This is based on prior service data: SD=5.5, baseline–follow-up correlation=0.30 and correlation among follow-ups=0.63. To account for unequal centre recruitment and clustering, a design effect of 1.336 was applied. Factoring in a 20% attrition rate, the final required sample size is 150. Power analysis was conducted using Stata 18 (StataCorp LLC, USA).

#### Statistical methods for primary and secondary outcomes

De-identified datasets will be downloaded from REDCap for analysis, which will follow an intention-to-treat (ITT) approach. Exploratory analyses of primary and secondary outcomes will precede formal analysis. Multilevel modelling will estimate treatment effects and their precision over follow-up, using participants as level 2 units, with baseline measures, minimisation factors, allocation, follow-up time and follow-up×allocation interaction as fixed-effect covariates.

The final SAP will detail planned ITT and sensitivity analyses for all outcomes. All trial data will be stored and analysed on secure University of Nottingham platforms using the latest version of Stata.

Missing outcome data will be assessed and reported by treatment group and timepoint. Multiple Imputation, assuming a Missing At Random (MAR) mechanism, will be used to address missing data. Sensitivity analyses will test the robustness of treatment effects against assumptions, limitations and analytical methods.

No interim analyses are planned.

#### Methods for additional analyses (eg, subgroup analyses)

### Qualitative analysis

A subsample of 20 participants, randomised to TPI+TAU and consenting to optional interviews, will be selected based on availability. These interviews will explore participants’ experiences of the TPI, its perceived impact and effects. If thematic saturation is not reached after 20 interviews, additional interviews will be conducted.

Semi-structured interviews will take place via telephone or Microsoft Teams after final follow-up. Anonymised transcripts will undergo Reflexive Thematic Analysis using Braun and Clarke’s six-step approach.^42^

### Economic evaluation

A within-trial economic evaluation will be conducted to assess cost-effectiveness from both health/social care and broader societal perspectives; full methods are detailed in online supplementary material 5.

### Oversight and monitoring

The CI has overall responsibility for the study and data custody, supported by the research team. The coordinating site, staffed by the University of Nottingham, manages trial operations. The Trial Management Group (comprising the CI, study researchers, trial manager and statistician) will meet fortnightly to monitor progress and address issues.

An independent NIHR ARC-EM Scientific Committee, external to the research team’s institutions, will review trial progress every 6 months. The study team will also meet with Patient and Public Involvement and Engagement (PPIE) members every 6 weeks to review recruitment and participant engagement.

The TSC/DMC, comprising a statistician, practitioner psychologist and PPIE representative, will provide independent oversight of the trial. Meeting at least every 6 months, the TSC/DMC will monitor trial conduct, participant safety, recruitment progress and data integrity, and will review recruitment and measure completion 6 months after initiation to assess feasibility. The committee will also review all reported AEs, which will be assessed for seriousness, expectedness and causality, and documented in participants’ records and case report forms (CRFs).

### Ethics and dissemination

Ethical approval for this trial was granted by the London - Bromley Research Ethics Committee (REC reference: 24/LO/0610) on 26 September 2024. The study will be conducted in accordance with Good Clinical Practice and the Declaration of Helsinki. All participants will provide informed consent before taking part.

Following participation, individuals can continue to access support for depression through their usual care pathways. Once discharged from EMCA CPH, participants will only be able to return to the service if formally re-referred by their clinical team and meeting the service’s standard eligibility criteria.

Dissemination will include sharing findings with participants who have consented to receive study results. In addition, results will be presented at national and international conferences, alongside publication in peer-reviewed journals. This document constitutes the full trial protocol. Requests for participant-level data or access to statistical code should be directed to the corresponding author. All protocol amendments will be submitted to the relevant ethics committee for approval. The sponsor and funder will also be notified, and the CI will ensure all study sites receive updated protocols for their Investigator Site Files. Any deviations will be documented using a breach report form, and the trial registry (https://www.isrctn.com/ISRCTN13692666) will be updated accordingly.

### Trial registry and status

The trial actively recruited via primary and secondary care NHS sites. Recruitment opened on 17 October 2024, and the first participant was recruited on 29 October 2024. Recruitment closed on 14 August 2025, by which time 160 participants had been recruited, exceeding the planned sample size of 150.

The trial was registered on ISRCTN on 18 October 2024: 13692666.

### Significance

The PROSPER trial addresses a critical and under-researched gap in psychological care for patients living with and beyond cancer who are experiencing depression while awaiting therapy, a period often marked by deterioration in mental health and leading to reduced engagement with services. This study evaluates the effectiveness of a brief, preparatory psychological intervention enhanced with personalised smart messaging, aiming to improve therapy readiness, adherence and clinical outcomes.

If found to be effective, the TPI could make a significant contribution to the evidence base supporting brief pre-treatment interventions for individuals awaiting psychological therapy. It would strengthen the rationale for incorporating such interventions into cancer care pathways. The integration of smart messaging represents an important innovation, with evidence that digital messaging can reinforce behavioural change, sustain motivation and increase patient activation, offering a low-cost, scalable adjunct to in-person care.^31 34^

From a clinical and health systems perspective, the findings of the PROSPER trial may inform future models of care delivery, particularly in oncology and other settings where there are delays in accessing behaviour change interventions. If the intervention is shown to be cost-effective and feasible to deliver by a range of trained staff, it could support service efficiency by reducing dropout rates and improving therapy outcomes. The pragmatic design of the trial, embedded within routine NHS care pathways, enhances the real-world relevance of the findings and supports the potential for widespread implementation.

### Patient and public involvement

We have consulted with three PPIE representatives (two with prior psychological therapy experience and one receiving cancer care) to inform the study’s relevance and impact. PPIE representatives have contributed to the study design, materials and recruitment strategy, ensuring alignment with patient needs and preferences. PPIE involvement will continue throughout the study, including data analysis and dissemination.

## Supplementary material

10.1136/bmjopen-2025-108442 online supplemental file 1
