# Supplementary material for: Preparation for online psychological therapy for depression in people living with and beyond cancer in East Midlands NHS primary and secondary care services in England: protocol for the PROSPER randomised controlled trial
Source: BMJ Open. 2026 May 18;16(5):e108442. doi: 10.1136/bmjopen-2025-108442 (PMC13185027; doi:10.1136/bmjopen-2025-108442)
Supplement: online supplemental file 1 [file bmjopen-16-5-s001.docx]

# Supplementary Material

## Supplementary material 1: PIS and Consent form


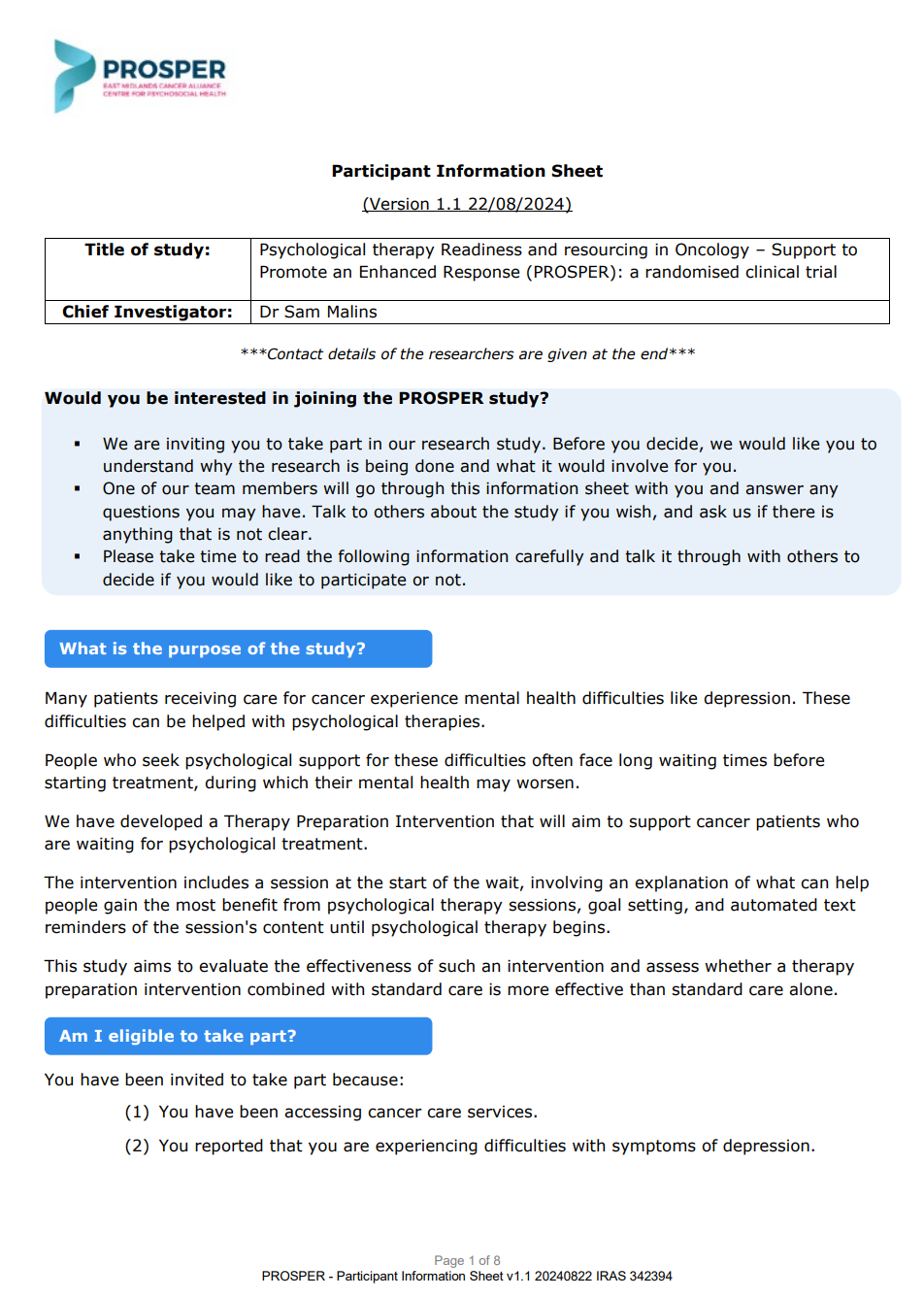


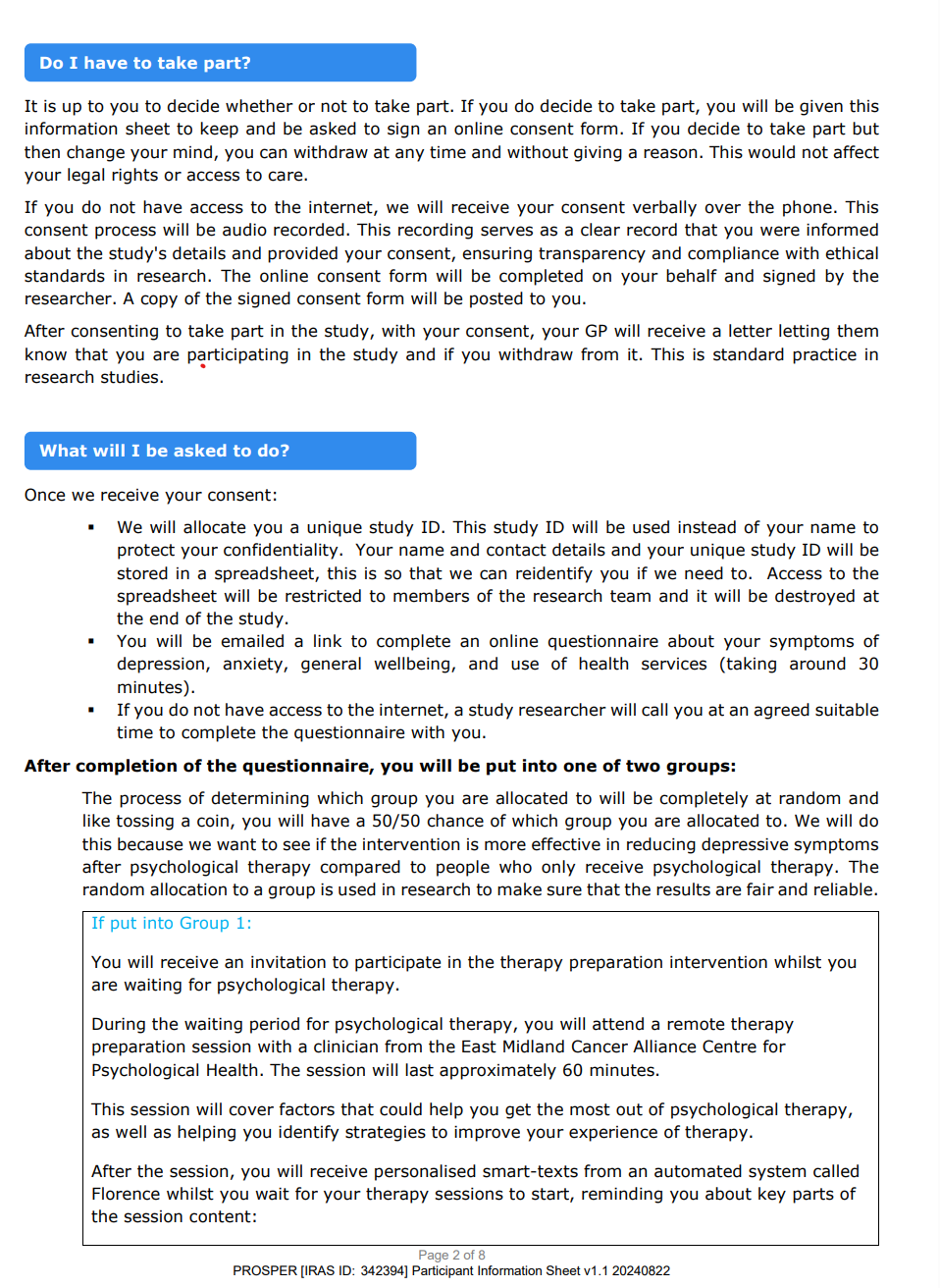


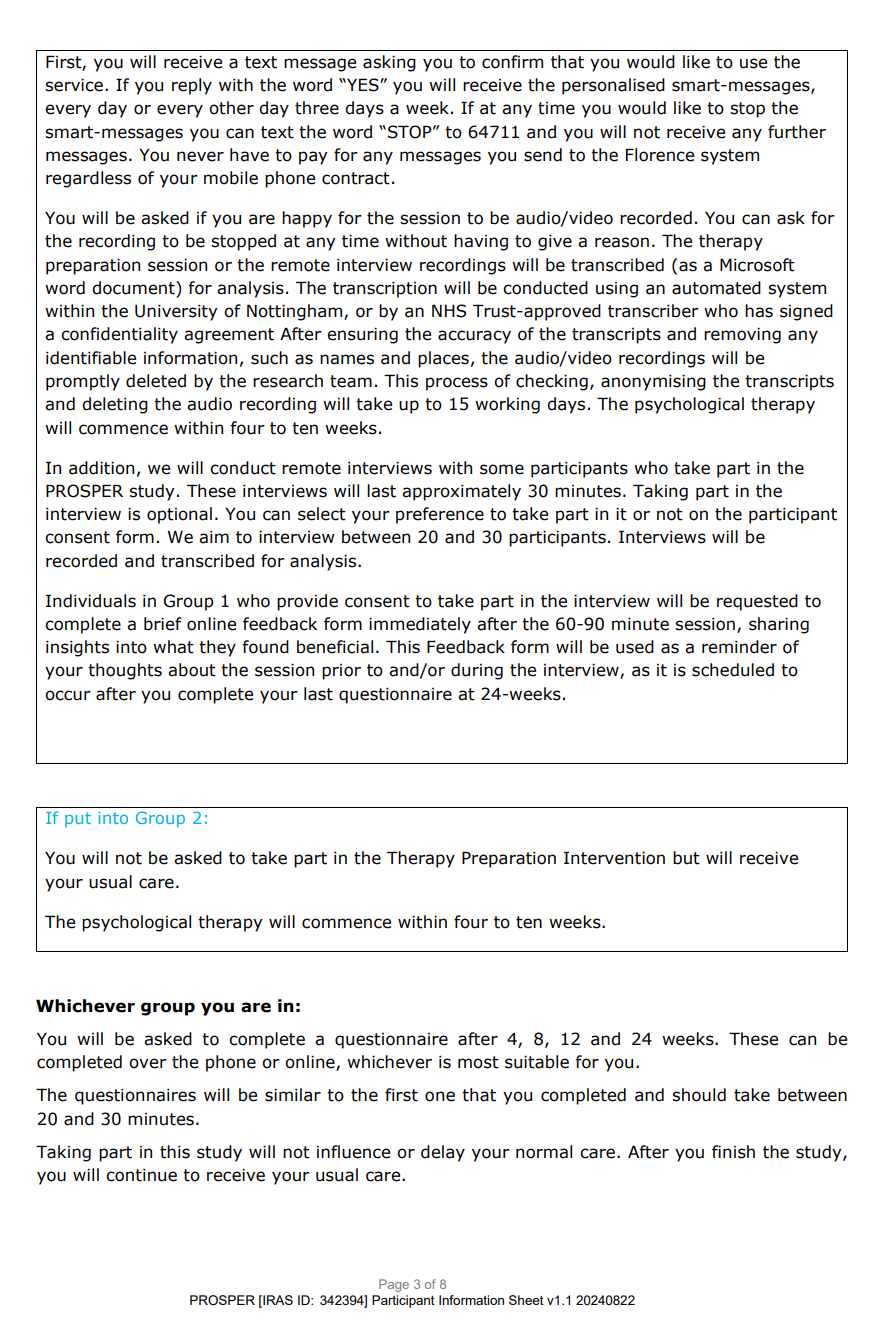


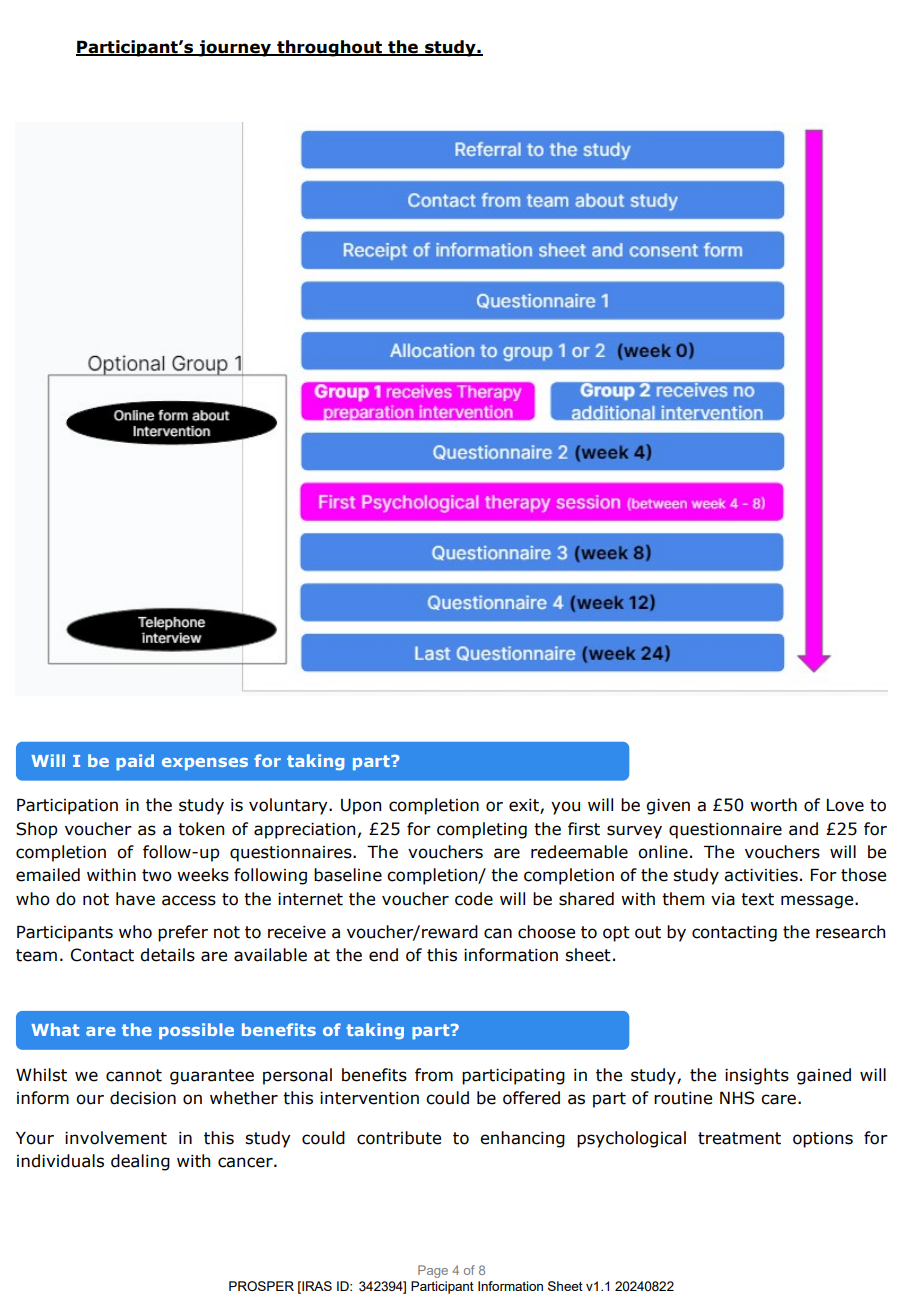


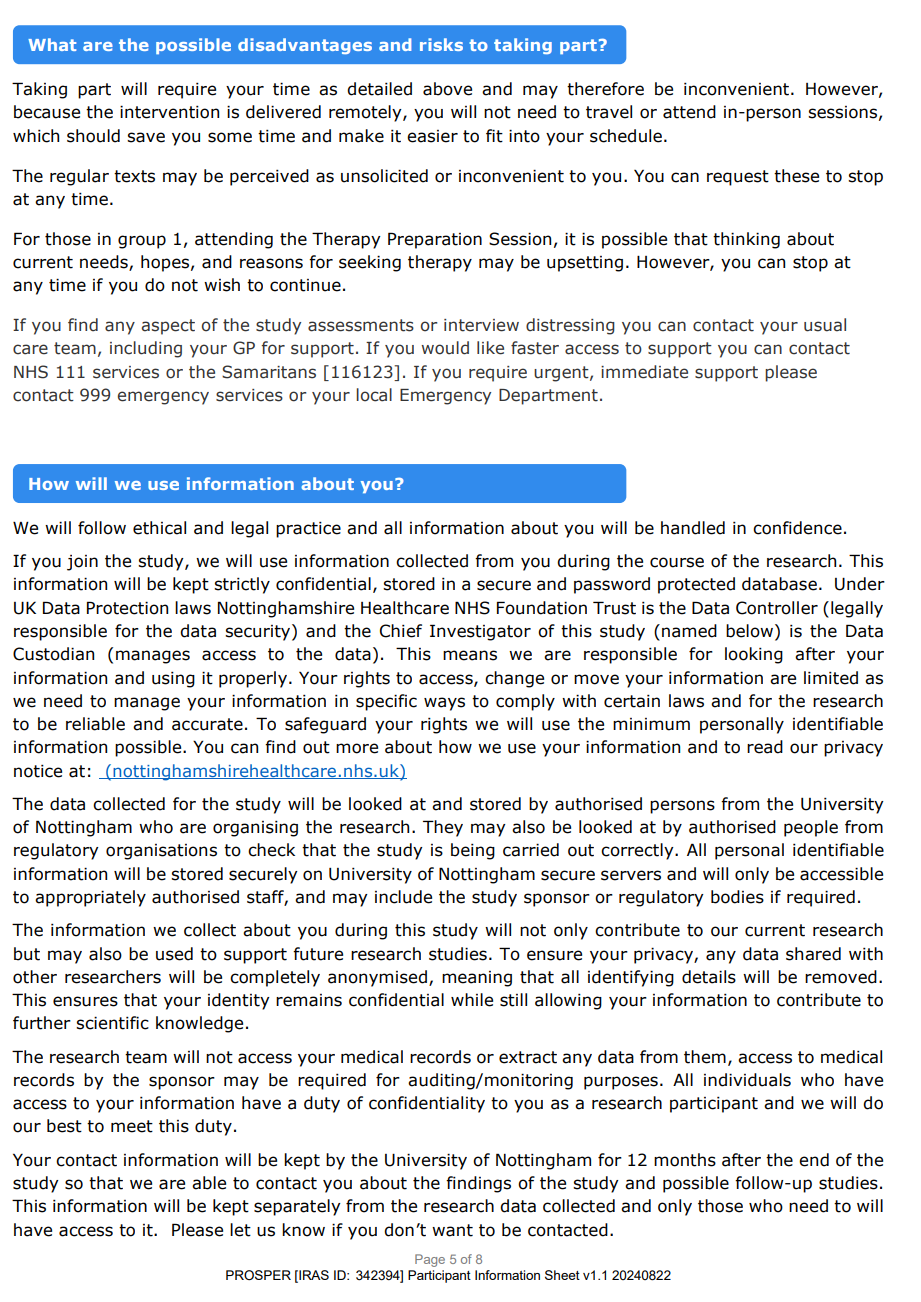


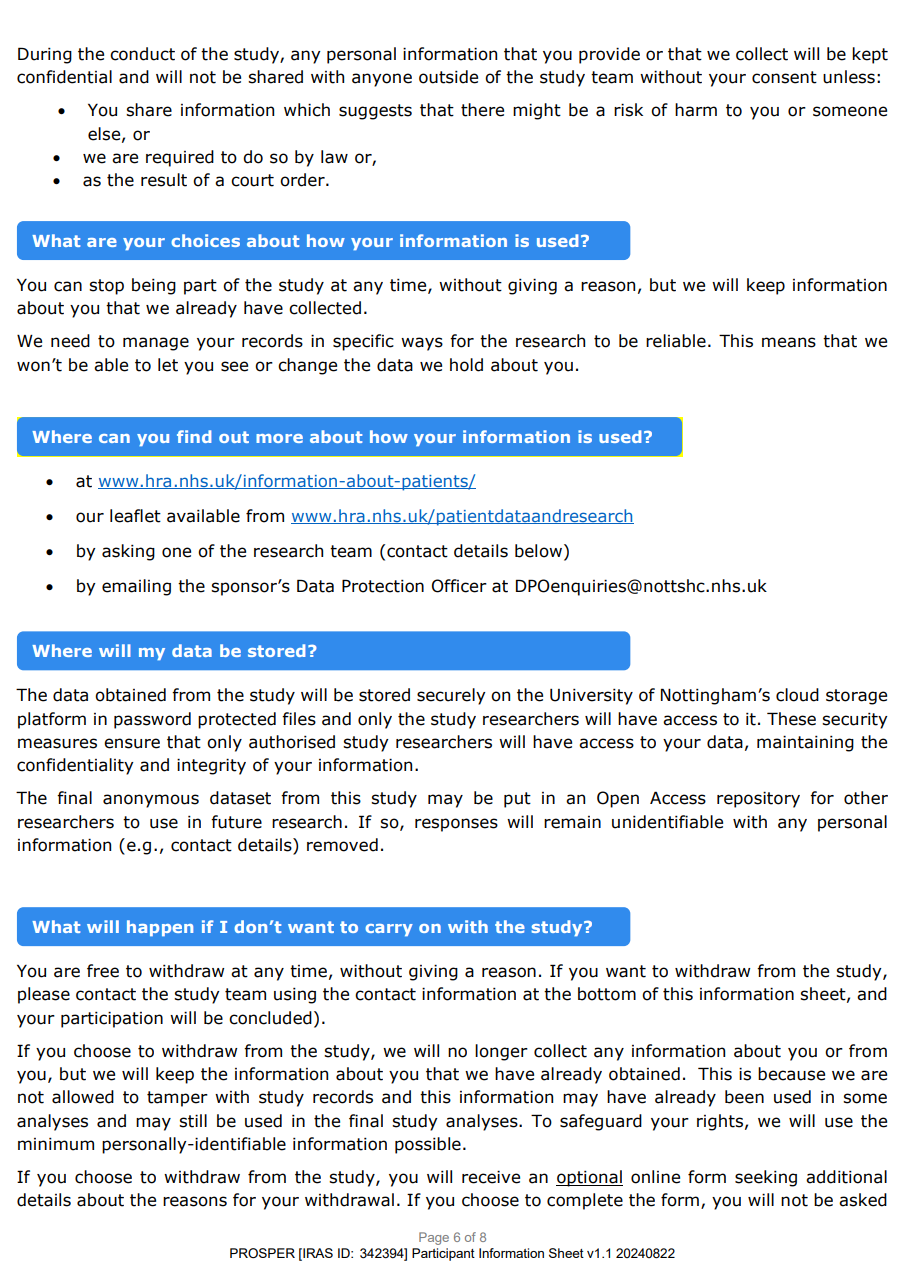


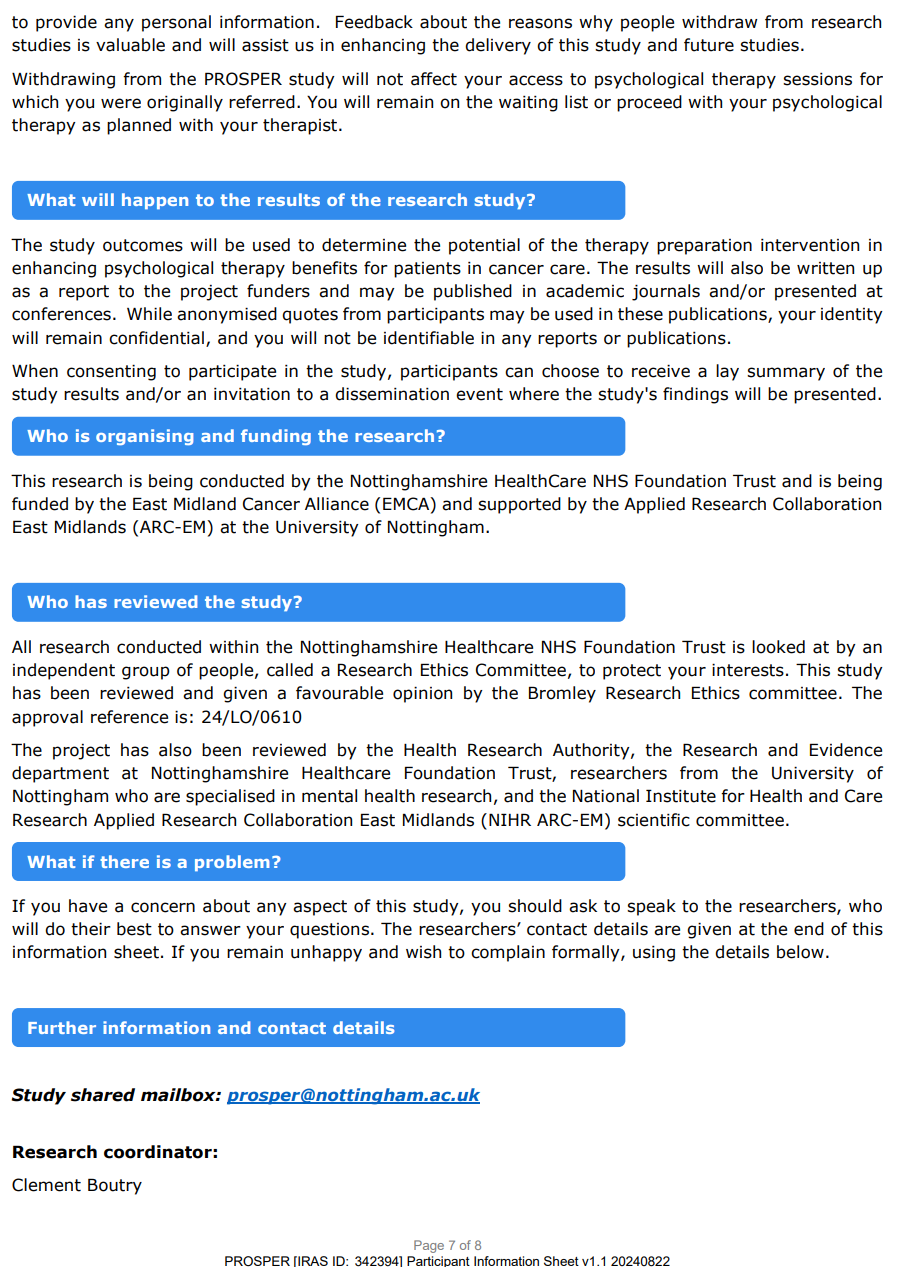


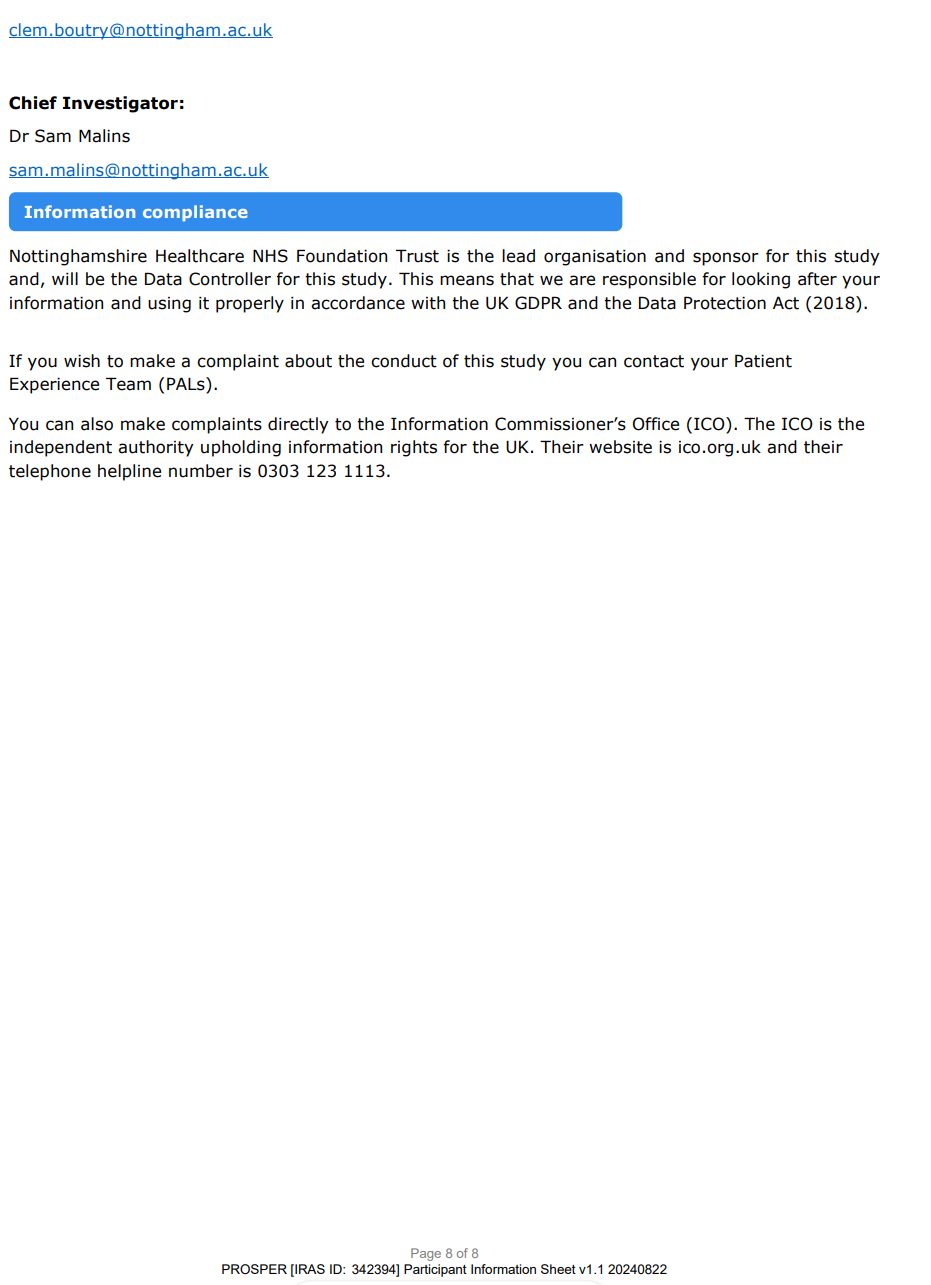


**CONSENT TO PARTICIPATE IN RESEARCH**

(Version 1.1 22/08/2024)

Please initial box

| 1 | I confirm that I have read the information sheet version 1.1 dated 22/08/2024 for the above study. I have had the opportunity to consider the information, ask questions and have had these answered satisfactorily. |  |
| --- | --- | --- |
| 2 | I understand that my participation is voluntary and that I am free to withdraw at any time without giving any reason, without my medical care or legal rights being affected. I understand that should I withdraw then any information collected about me up to that point will be kept in line with the Participant Information Sheet and that the information I have provided may still be used in the project analysis. |  |
| 3 | I understand that relevant sections of my medical notes and data collected during the study, may be looked at by authorised individuals from Nottinghamshire Healthcare NHS Foundation Trust, the study team, or from regulatory authorities, where it is relevant to me taking part in this study. I give permission for these individuals to have access to relevant information in my medical records and to collect, store, analyse and publish information obtained from my participation in this study.  I understand that any publications will not contain any information that could be used to identify me. |  |
| 4 | I agree to my GP being informed of my participation in this study and to be contacted if my responses to the study questionnaires raise concerns about my wellbeing. |  |
| 5 | I understand that the information collected about me will be used to support   other research in the future and may be shared anonymously with other researchers. |  |
| 6 | I agree for any treatment sessions and interviews conducted during my participation to be audio/video recorded and transcribed into written form by the researchers or a transcription service approved by the study sponsor. I understand that the original recordings will be deleted after they have been transcribed and checked for accuracy. |  |
| 7 | I agree to receive personalised smart text messages, I understand that it is an automated service that cannot be used to seek healthcare and I am aware that messages can be stopped at any time by texting STOP to 64711. |  |
| 8 | **Optional:**I would like to receive a summary of the results of the study.  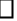 Yes           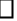 No |  |
| 9 | **Optional:** I would like to participate in the recorded remote interview.  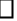 Yes           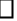 No |  |
| 10 | **Optional:** I would like to be invited to a result dissemination event where the findings of the study will be presented.  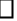 Yes           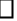 No |  |
| 11 | I agree to take part in the above study. |  |

Please initial box

Name of Participant Date Signature

Name of Person taking consent Date Signature

## Supplementary material 2: Treatment as Usual (TAU)

In the host service, TAU involves receiving psychological therapy via video call or telephone for depression (with or without anxiety) and includes a typical waiting period of 4–12 weeks from the point of referral. Therapy is delivered by a practitioner psychologist following an integrative approach, drawing primarily on contextual Cognitive Behavioural Therapy processes (with some variability between therapists). On average, the psychological therapy lasts for 12 weeks.

At the start of the waiting period for psychological therapy, all patients are provided with a document aimed at helping them gain the most benefit from their therapy sessions. This document encompasses an overview of the service, expectations for sessions, an introduction to confidentiality and privacy concepts, and practical guidelines. These practicalities include session preparation tips such as checking connections, being ready for calls in advance, and guidance on addressing potential technical difficulties.

## Supplementary material 3: Treatment Preparation Intervention

All participants in the TPI arm of the trial will receive this intervention during the waiting period in addition to TAU (i.e., standard psychological therapy and pre-therapy document).

The TPI will be provided to participants on an individual basis, as (1) a video or telephone consultation with a practitioner for up to 60 minutes, followed by (2) personalised SMS-based smart-messaging (delivered via an automated messaging system).

For cancer patients awaiting psychological therapy for psychological distress, a brief TPI may help to foster hope and readiness and enable them to make more active use of subsequent therapy, which may lead to greater therapeutic gains.

**Therapy Preparation Session**

The Therapy Preparation Session (TPS) is a single 60-minute session delivered within two weeks of referral to the host service. Facilitated by a trained practitioner psychologist, the session encompasses Motivational Interviewing techniques to enhance engagement with psychological therapy (S. D. Miller et al., 2023; W. R. Miller & Rollnick, 2002). Participants are offered optional pre-session questionnaires (the short-form URICA and Signature Strengths Survey) to personalise the session to their individual presentation on the basis of their readiness for change and key character strengths that could be drawn upon for therapeutic benefit (Mander et al., 2012; McGrath, 2019). These questionnaires will be linked to other assessments at the end of the study in a mechanisms of action analysis.

The TPS incorporates psychoeducation on therapy expectations, therapist and patient roles, and behaviour change principles. Patients’ treatment preferences are elicited and accommodated, where possible. Key motivations and an initial action plan are elicited in the form of:

1. Patients’ hopes and reasons for attending therapy,
2. Patients’ existing strengths, skills and strategies that may be preventing deterioration in their mental health
3. First steps to change that could be taken whilst awaiting the start of therapy, particularly those that build on established healthy coping strategies and strengths.

After the session, the motivations and action plan are then sent to patients as reminder messages throughout the waiting period.

For the TPS, session flow and duration of components will be flexibly adjusted on a case-by-case basis, but core activities (and approximate timings) will be:

5 minutes: **Introduction**

- Troubleshoot any connection problems
- Set context of referral (e.g. you’ve been referred by…)
- Check able to speak in a private space
- State purpose of call and set expectations (e.g. Not an assessment –some information about what to expect from sessions, and what might help you get the most from them)
- Statement of limits of confidentiality

5 minutes: **Elicit treatment preferences:**

- Identify preferred timing and other practicalities of session scheduling
- Preferred characteristics of therapist
- Explaining that these will be passed on to their treating therapist

10 minutes: **Psychoeducation:**

- Role induction
  - Therapy length and format
  - “Jobs” of therapist and patient (e.g. talking, structuring and guiding the session)
  - Importance of giving and receiving feedback
  - Identifying one or more “helpers/supports” outside therapy
  - Engaging with activities between sessions – homework tasks
- Hope and expectancy
  - How and why psychological therapy works
  - Clarify the likelihood of improvement and recovery from difficulties following psychological therapy
  - Expected rate of change
  - What to do if little or no change ensues

10 minutes: **Motivations** **Discussion (Reasons):**

- - The patient’s main hopes, needs, reasons, and desires that they would like psychological therapy to help them with

10 minutes: **Motivations Discussion (Strategies):**

- - Obstacles that may prevent them from achieving their hopes
  - Their best strategies for addressing these obstacles and getting the most from psychological therapy. This can be informed by information on the strategies used by previous patients and previous messages used.
  - Pass on advice from previous patients or show videos of testimonials

10 minutes: **Motivations Discussion (First steps):**

- - The first steps towards achieving their hopes or addressing an obstacle that could be started within the next day (e.g. “Given our discussion today what, if anything, would you like to do differently in the period while your waiting for your sessions to start?”)
  - Discussion sections above are interweaved with the patients character strengths, how they could leverage them in pursuit of their hopes for therapy and overcome obstacles.

5 minutes: **Session close down**:

- - Explanation of their next contact
  - Possible length of wait
  - What to do if there are any problems
  - Check in that signed up and able to receive Therapy Preparation Messaging
  - Address any final questions

**Therapy Preparation Messaging**

Following the TPS, participants will receive personalised text messages sent via the Florence automated messaging platform, reminding them of key points from the TPS. Participants will receive three messages per week, including a message 1) reminding participants for their reasons to attend therapy (“This is a reminder that you are going to therapy to help improve your relationship with your family”), 2) reminding participants of strategies that they think will help them maintain engagement with therapy (“This is a reminder that writing things down and putting them on the fridge often helps you remember to get things done”), and 3) reminding participants of first steps to helpful change that they identified at the TPS (“This is a reminder that a first step you could take to help yourself would be to go for a 10 minute walk after breakfast in the park”). Participants will continue to receive messages for six weeks after the TPS. Participants will be able to stop receiving texts by texting STOP at any time. Participants’ data collected via text messages (text messages including interactions with the Florence system, last digits of their phone number) will be kept on the Florence system until the end of the analysis.

## Supplementary Material 4: Objectives, measures and timepoints summary

| **Objectives** | **Outcome Measures** | **Timepoint(s) of evaluation of this outcome measure (if applicable)** |
| --- | --- | --- |
| **Primary Objective**  To compare change in depression score between the TPI and TAU. | Patient Health Questionnaire 9- items (PHQ-9) | 4-, 8-, 12-, and 24-week follow-up assessments |
| **Secondary Objectives (clinical effectiveness)**  To compare change in additional clinically significant outcomes including anxiety, quality of life, mental wellbeing, and utilisation of health services, between the TPI and TAU. | Generalised Anxiety Disorder assessment 7-items (GAD-7),  Work and Social Adjustment Scale (WSAS)  Short Warwick-Edinburgh Mental Wellbeing Scale (SWEMWBS)  EuroQol 5-Dimensions 5-Levels (EQ5D5L) | 4-, 8-, 12-, and 24-week follow-up assessments |
| **Secondary outcomes (mechanism of action)**  To explore the mechanism of action of the TPI and TAU. | Patient Activation Measure (PAM)  Beck Hopelessness Scale  Readiness for Change Ruler | 4-, 8-, 12-, and 24-week follow-up assessments |
|  | Consultation Interactions  Coding Scheme (CICS) | Recording of the TPS |
| Service usage monitoring | Client Service Receipt Inventory (CSRI) | 24 weeks follow-up assessment |
| To assess differences in patient engagement between the TPI and TAU. | Treatment dropout  Session non-attendance | Review of attendance record at 24-week follow-up |

## Supplementary material 5: details of the economic evaluation

A within-trial economic evaluation will be undertaken from a health and social care perspective in the base case, with a broader societal perspective considered in secondary analyses in order that impacts on the family, work and benefits are captured in addition to health and social care impacts. Resource use incurred by the NHS, social services, families and employers will be collected using an adapted version of the Client Service Receipt Inventory (CSRI, (Beecham & Knapp, 2001). The CSRI is completed through interviews with the participant collected at baseline, 4-, 8-, 12- and 24-weeks follow-up.  These will be costed using published unit costs, for example, from the Personal Social Services Research Unit (PSSRU, 2019). NHS reference costs and British National Formulary. The outcome measure used for the economic analysis will be quality-adjusted life years (QALYs), derived from utility scores obtained using the EuroQoL EQ-5D-5L instrument. This instrument has demonstrable responsiveness to changes in depression, even over short time periods (Gerhards et al., 2011). Utility values will be calculated using the tariff recommended at the time of analysis (NICE, 2019). Following NICE recommendations, utility values in reference-case analyses will be calculated by mapping the 5-level descriptive system data onto the 3-level value set (NICE, 2019). Using this information on costs and benefits, an incremental cost utility analysis will be conducted and reported using accepted methodology, including a cost-effectiveness acceptability curve showing the probability that the intervention is cost-effective at a range of threshold values for the willingness to pay per QALY (Ramsey et al., 2015).

## Supplementary Material 6: Statistical Analysis Plan

Psychological Therapy Readiness and Resourcing In Oncology – Support to Promote An Enhanced Response (PROSPER): A Randomised Controlled Trial

Statistical Analysis Plan

Final version 1.0

(27^th^ February 2026)

Based on Protocol version(3) 2.0 (dated 26^th^ February 2025)

Trial registration(1): ISRCTN 13692666

| The following people have reviewed the Statistical Analysis Plan and are in agreement with the contents_(6)_ | | | |
| --- | --- | --- | --- |
| **Name** | **Job title** | **Trial Role** | Date |
| Clement Boutry | Research Associate | Trial researcher | 23/02/2026 |
| Paulina Hagyari-Donaldson | Research Assistant | Trial researcher | 23/02/2026 |
| Boliang Guo | Statistician | Trial  Statistician | 18/01/2026 |
| Sam Malins | Consultant Clinical Psychologist | Chief Investigator | 13/02/2026 |
| Shireen Patel | Research Fellow | Trial Manager | 13/02/2026 |
| Nima Moghaddam | Professor of Clinical & Health Psychology | Psychological Therapy process expert | 13/02/2026 |
| Richard Morriss | Professor of Psychiatry | NIHR mentor | 27/02/2026 |

***Note: please keep all subscript numbers such as*** (6) ***in the document, they are indexing number for content in JAMA SAP templates, DO NOT REMOVE THEM. I will remove this note in final version SAP.***

| **Abbreviations** | **Description** |
| --- | --- |
| AE | Adverse Event |
| AR | Adverse Reaction |
| CI | Chief Investigator |
| CRF | Case Report Form |
| CRN | Clinical Research Network |
| CSRI | Client Service Receipt Inventory |
| DMC | Data Monitoring Committee |
| GAD-7 | Generalised Anxiety Disorder – 7 items |
| GCP | Good Clinical Practice |
| ICF | Informed Consent Form |
| MI | Motivational Interviewing |
| NHS R&D | National Health Service Research & Development |
| PIS | Participant Information Sheet |
| QA | Quality Assurance |
| QC | Quality Control |
| PHQ-9 | Patient Health Questionnaire – 9 items |
| RCT | Randomised Control Trial |
| REC | Research Ethics Committee |
| SAE | Serious Adverse Event |
| SAR | Serious Adverse Reaction |
| SDV | Source Data Verification |
| SOP | Standard Operating Procedure |
| SUSAR | Suspected Unexpected Serious Adverse Reaction |
| SWEMWBS | Short Warwick-Edinburgh Mental Well-being Scale |
| TAU | Treatment As Usual |
| TMF | Trial Master File |
| TMG | Trial Management Group |
| TPI | Therapy Preparation Intervention |
| TPM | Therapy Preparation Messages |
| TPS | Therapy Preparation Session |
| TSC | Trial Steering Committee |
| WSAS | Work and Social Adjustment Scale |

**Additional contributors to the SAP (non-signatory)** _(5)_

| **Name** | **Trial role** | **Job Title** | **Affiliation** |
| --- | --- | --- | --- |
|  |  |  |  |
|  |  |  |  |
|  |  |  |  |

# **Introduction**

This document details the rules proposed and the presentation that will be followed (Gamble et al., 2017) as closely as possible, when analysing and reporting the main results from the study titled “*Psychological Therapy Readiness and Resourcing In Oncology – Support to Promote An Enhanced Response (PROSPER): A Randomised Controlled Trial*”. These analyses will assess the efficacy and safety of a Therapy Preparation Intervention (TPI) plus Treatment As Usual (TAU) in comparison with the TAU and will be included in the clinical study report.

The purpose of the plan is to:

- Ensure that the analysis is appropriate for the aims of the trial, reflects good statistical practice, and that interpretation of a priori and post hoc analyses respectively is appropriate.
- Explain in detail how the data will be handled and analysed to enable others to perform or replicate these analyses.

Additional exploratory or auxiliary analyses of data not specified in the protocol may be included in this analysis plan. This analysis plan will be made available if required by journal editors or referees when the main papers are submitted for publication. Additional analyses suggested by reviewers or editors will be performed if considered appropriate. This should be documented in a file note.

Amendments to the statistical analysis plan will be described and justified in the final report of the trial and where appropriate in publications arising from the analysis. Health economic and qualitative analysis plans are beyond the scope of this document.

## **Background and rationale** _(7)_

Depression and anxiety are prevalent among people living with cancer/undergoing cancer treatment compared to the general population, with estimates suggesting that psychological morbidity often remains under-recognised and under-treated (Forbes et al., 2024; Pitman et al., 2018). Difficulties with mental health in this population are associated with worse treatment adherence, increased use of healthcare services, and reduced survival (DiMatteo & Haskard-Zolnierek, 2010). Moreover, delays in accessing psychological support may exacerbate distress, increase dropout rates, and reduced treatment efficacy (Carter et al., 2012; Furukawa et al., 2014; Reitzel et al., 2006).

Standard care via NHS Talking Therapies often involves substantial waiting periods (NHS digital, 2024), during which distress may worsen (Steinert et al., 2017). There is growing interest in brief, scalable interventions that can mitigate distress and enhance engagement during these delays (Singla et al., 2023).

One promising area of innovation in mental health care is the development of structured, single-session interventions. These brief formats have demonstrated effectiveness across a range of populations and clinical issues, offering immediate symptom relief, increased motivation, and enhanced engagement with ongoing treatment (Cameron, 2007; Schleider et al., 2025). Their brevity and scalability make them particularly appealing in contexts where service access is delayed or resource-limited.

Digital technologies, such as automated messaging systems and app-based supports, are increasingly being used to extend the reach and reinforce the impact of psychological interventions. These tools can deliver tailored reminders, motivational content, and behavioural prompts, helping to consolidate therapeutic gains and maintain engagement during periods of risk or delay (Malins et al., 2020; Wells et al., 2020).

Building on this, a TPI was developed, tailored to patients with an existing cancer diagnosis awaiting psychological support. The TPI comprises two components: (1) a structured, single-session psychological intervention informed by motivational interviewing and cognitive behavioural principles, and (2) a six-week programme of automated smart messaging, designed to echo and reinforce session content. This integrated approach aims to provide immediate support while promoting readiness and sustained engagement with subsequent therapy.

This trial evaluates the clinical effectiveness of TPI + TAU versus TAU alone in reducing depressive symptoms in people with a diagnosis of cancer referred to NHS Talking Therapies.

This SAP specifies the pre-defined analytical methods for evaluating the primary and secondary outcomes of the trial, in accordance with CONSORT guidelines. The SAP was written prior to database lock to minimise bias and ensure transparency of reporting

## **1.2 Objectives** _(8)_

**Study objectives**

The overall aim is to assess the effectiveness of a TPI for adult cancer patients awaiting psychological therapy, in terms of its effects on clinically important outcomes such as depression (primary outcome), anxiety, and therapy dropout.

Primary objectives

The primary objective is to assess the clinical effectiveness of TPI+TAU vs. TAU in change on PHQ-9 from baseline across 4-, 8-, 12, and 24-week follow-up weeks post randomisation.

Secondary objectives

The secondary objectives are to assess the clinical effectiveness of TPI+TAU vs. TAU in change on secondary outcomes from baseline across 4-, 8-, 12, and 24-week follow-up weeks post randomisation. The secondary outcomes include: GAD-7, WSAS, SWEMBS, PAM, BHS4, and a three items readiness for change ruler. In addition, health-related quality of life will be assessed at 24 weeks using the Visual Analogue Scale (VAS) of the EQ-5D-5L.

**Research hypothesis**

The null hypothesis for primary outcome is that there is no difference in change on depression from baseline across follow-ups post randomisation between the two groups. The alternative hypothesis is that there is a difference between the two groups.

# **Study methods**

## **2.1 Trial design** _(9)_

This study is a two-arm, multicentre, single-blind randomised controlled trial comparing TPI + TAU (Treatment as Usual) with TAU alone across 24-week follow-up for moderate-to-severe depression among cancer care patients in the East Midlands. Participants will be invited from patients being seen in cancer care across the East Midlands, who have been referred to the service hosting the trial and are awaiting psychological therapy for moderate-to-severe depression. Participants will be randomised in a 1:1 ratio to TPI + TAU or TAU alone. Follow-up assessments will be conducted at 4-, 8-, 12- and 24-week post randomisation. Qualitative analysis of semi-structured interviews of participants receiving TPI + TAU will be nested in the study.

## **2.2 Randomisation & Blinding** _(10)_

Randomisation will be conducted through REDCap which is a web-based system established by the University of Nottingham Clinical Database Support Service. Information required for randomisation of participants will include the participant ID, the centre, and their GAD-7 score (i.e., ≥8; moderate anxiety). Randomisation data will be password-protected and only accessible by an unblinded trial coordinator (study administrator from EMCA CPH) or their nominee. Participants will be individually randomised in a 1:1 ratio to either TPI + TAU or TAU alone, with minimisation based on the participant centre and on a moderate and above level of anxiety (i.e., scores ≥8) measured by GAD-7. Although the primary outcome focuses on depressive symptoms this minimisation emphasis on anxiety is due to the intervention including and aiming to support both those with depressive symptoms alone and those with both anxiety and depressive symptoms, given that patients with comorbid anxiety and depression typically have a lower response to treatments for depression than those with depression alone (Andreescu et al., 2007). EMCA CPH administrators will communicate treatment allocation to participants, whilst also notifying TPI-delivering clinicians.

Participants will have completed their baseline assessment and be randomised within one week (seven days) of receiving their informed consent. For those randomised to TPI + TAU, the TPS will be scheduled within one week of randomisation. Researchers completing trial assessments and trial statistician will be blinded to arm allocation.

## **2.3 Sample size** _(11)_

Based on previous patient-centred work estimating minimally clinically important difference (MCID) on the PHQ-9, we would expect MCID to be equivalent to a difference of 3 points (i.e., ~20% of baseline severity seen in our previous service data, where baseline M = 16.3) (Morriss et al., 2016). A target MCID of 3 points is consistent with estimation in previous trials of psychological therapy for depression in cancer patients. To detect a 3-point difference in PHQ-9 scores in the present study, 90 participants will be required to achieve 90% power at a two-tailed significance level of 0.05. This is based on estimates from previous EMCA CPH service data for the average SD (5.5 across timepoints), assuming equal SD for both groups, correlation between baseline and follow-ups of 0.30, and correlation among follow-ups of 0.63. Given the unknown unequal allocation rate from each recruitment centre and possibility of cluster design for some centres (about 10 ~ 15 potential recruitment centres) and reference on clustering effects (ICC = 0.1) from a previous RCT (Vierron & Giraudeau, 2019), a design effect =1.336 was chosen to inflate the requested sample size. After adjusting a 20% loss to follow-up rate, a required sample size of 150 in total was calculated. Stata sampsi code was used to perform the power analysis.

## **2.4 Framework**_(12)_

In line with the study objectives which was specified in the trial protocol and reiterated here in section 1.2, both primary and secondary outcomes are testing for superiority of the treatment effectiveness of the TPI+TAU over TAU.

## **2.5 Statistical interim analyses and stopping guidance** _(13)_

There is no formal interim analysis planed.

## **2.6 Timing of final analysis** _(14)_

The dataset will be locked for final analysis once the last recruited participant’s last follow-up outcome data is available.

## **2.7 Timing of outcome assessments** _(15)_

The schedule of study procedures for all data collection is given in the Table 1 at section 5.1. Briefly, both primary and secondary outcome measures will be collected at baseline and at 4-, 8-, 12-, and 24- weeks post randomisation.

# **Statistical Principals**

## **3.1 Confidence intervals and P values** _(16-18)_

All applicable statistical tests will be 2-tailed and will be performed using a 5% significance level; There is no planned adjustment for multiplicity as the study has only one primary outcome (EMA, 2016). All confidence intervals presented will be 95% and 2-tailed.

## **3.2 Adherence and protocol deviations** _(19)_

Adherence is defined as the extent to which participants engage with the prescribed components of the TPI, including attendance at the TPS, and ongoing participation in psychological therapy. This will be assessed through dropout rates, and TPS and session attendance over the course of therapy.

Protocol deviations are classified prior to unblinding of treatment. The number (and percentage) of participants with major and minor protocol deviations will be summarised by treatment group with details of type of deviation provided. The participants that are included in the Intention To Treat (ITT) analysis data set will be used as the denominator to calculate the percentages. The number (and percentage) of participants with major and minor protocol deviations will be summarised by treatment group with details of type of deviation provided. The participants that are included in the ITT analysis dataset will be used as the denominator to calculate the percentages. No formal statistical testing will be undertaken.

## **3.3 Analysis populations** _(20)_

The ITT population will include all randomised participants, regardless of their eligibility, according to the treatment they were randomised to receive.

A per-protocol population (PPA) will be considered for per-protocol analysis as one sensitivity analysis.

# **Trial population**

## **4.1 Screening data** _(21)_

The number of participants screened will be presented in CONSORT flow diagrams (figure 1).

## **4.2 Eligibility** _(22)_

Inclusion criteria:

- Aged 18 years or older;
- Able to engage with psychological therapy sessions conducted in English;
- Competent to give informed consent;
- Diagnosed with cancer and awaiting psychological therapy with EMCA CPH for symptoms of moderate-to-severe depression during the recruitment period of the study;
- A score of 10 or more on PHQ-9.

Exclusion criteria:

- Immediate risk to self or others;
- Currently receiving psychological therapy with another service;
- Unable or unwilling to receive care remotely.

The consort diagram will report the numbers and reasons for non-inclusion to the trial prior to randomisation and also report any withdrawals post-randomisation together with reasons if any.

## **4.3 Recruitment** _(23)_

A CONSORT flow diagram (figure 1) will be used to summarise the number of participants who were:

- assessed for eligibility at screening
  - eligible at screening
  - ineligible at screening*
- eligible and randomised
- eligible but not randomised*
- received the randomised allocation
- did not receive the randomised allocation*
- lost to follow-up*
- discontinued the intervention*
- randomised and included in the primary analysis
- randomised and excluded from the primary analysis*

*Reason will be provided

## **4.4 Withdrawn/follow-up** _(24)_

The level of consent/withdrawal will be tabulated (classified as “consent to continue follow-up and data collection”, “consent to continue data collection only”, “complete – no further follow-up or data collection”). This will be presented in CONSORT diagram format rather than as a table, with numbers and reasons for withdrawal and/or exclusion from analysis given at each stage.

Analysed (n= )
♦ Excluded from analysis (give reasons) (n= )

Lost to each follow-up (give reasons) (n=)

Discontinued intervention (give reasons) (n= )

Allocated to intervention (n= )

♦ Received allocated intervention (n= )

♦ Did not receive allocated intervention (give reasons) (n= )

Lost to each follow-up (give reasons) (n=)

Discontinued intervention (give reasons) (n= )

Allocated to intervention (n= )

♦ Received allocated intervention (n= )

♦ Did not receive allocated intervention (give reasons) (n= )

Analysed (n=)
♦ Excluded from analysis (give reasons) (n=)

Allocation

Analysis

Follow-Ups

Assessed for eligibility

Excluded (n= )

♦  Not meeting inclusion criteria (n= )

♦  Declined to participate (n= )

♦  Other reasons (n= )

Randomized (n=)

Enrolment

Figure 1: Example of skeleton CONSORT flow diagram

## **4.5 Baseline participant characteristics** _(25)_

Participants will be described with respect to demographic data (age, sex, ethnicity, marital status, highest level of education, presence of disability, religion, and first language); The details of descriptive statistics are reported in 5.2.1. Tests of statistical significance will not be undertaken for baseline characteristics; rather the clinical importance of any imbalance will be noted (European Medicines Agency, 2013).

# **Analysis**

## **5.1 Outcome definitions** _(26)_

**Table 1:** Summary of the outcome measures

| Outcome measures | Scale, description  and source | Derivation of scores |  | Time point (week) | | | |
| --- | --- | --- | --- | --- | --- | --- | --- |
|  |  |  |  |  |  |  |  |
| Primary outcome |  |  | 0 | 4 | 8 | 12 | 24 |
| Change from baseline on PHQ-9 across 4, 8,12 and 24 weeks. | The self-rated Patient Health Questionnaire for depression (PHQ-9) has 9 items measuring depressive symptomology. Each item asks the individual to rate how often they have been bothered by the listed symptoms over the past two weeks, with responses ranging from 0 (not at all) to 3 (nearly every day). | The total score is calculated by summing the item response, range from 0-27 with higher score indicating higher level of depressive severity. |  |  |  |  |  |
| Secondary outcomes |  |  |  |  |  |  |  |
| Change from baseline on GAD-7 across 4, 8,12 and 24 weeks. | Generalised Anxiety Disorder (GAD-7) is a 7-item instrument used to measure or assess the severity of Generalised Anxiety Disorder. Each item asks the individual to rate the severity of their symptoms over the past two weeks. Response options range from 0 (not at all) to 3 (nearly every day). | The total score is calculated by summing the item response, range from 0-21 with higher score indicating higher level of anxiety severity. |  |  |  |  |  |
| Change from baseline on Work and Social Adjustment Scale (WSAS) across 4, 8,12 and 24 weeks. | to assess the impact of mental health problems on an individual's ability to perform day-to-day activities and fulfil social roles through five items (“ Because of my condition -here, mental health, my ability to work is impaired”) scored between 0 and 8 giving a total score ranging from 0 (no impairment) to 40 (severe impairment) | The WSAS comprises five items, each rated on a scale from 0 (not at all impaired) to 8 (very severely impaired). The total score ranges from 0 to 40, calculated by summing the scores of all items. ​  Interpretation:  Scores above 20 indicate moderately severe impairment or worse psychopathology.​  Scores between 10 and 20 suggest significant functional impairment but less severe clinical symptomatology.​  Scores below 10 are considered subclinical or indicative of low impairment  Over study recruitment, data quality monitoring revealed that approximately half of participants were not working, for reasons unrelated to their health (e.g., retired). Therefore, Item 1 of the Work and Social Adjustment Scale (WSAS; work/study) was not applicable to them. To address this,  a pro-rated scoring approach was applied to calculate a 0–40 comparable WSAS total. This scoring method has been used effectively in recent studies (Adamson et al., 2020; Suh et al., 2025). |  |  |  |  |  |
| Change from baseline Short Warwick-Edinburgh Mental Wellbeing Scale (SWEMWBS) across 4, 8,12 and 24 weeks. | to measure general mental wellbeing through seven items (e.g., “I have been feeling optimistic about the future”) rated on a scale from 1 to 5 leading to total scores ranging from 7 (low mental wellbeing) to 28 (high mental wellbeing) | SWEMWBS consists of seven positively worded items, each scored from 1 to 5. The total raw score ranges from 7 to 35. For accurate interpretation, raw scores are transformed into metric scores using a conversion table. ​  Interpretation: Higher scores reflect greater positive mental wellbeing. While specific categorizations may vary, comparing individual scores to national survey data can provide context. |  |  |  |  |  |
| Change from baseline on EuroQol 5-Dimensions VAS across 24 weeks. | to measure health related quality of life, assessing five dimensions including mobility, usual activities, pain/discomfort and anxiety/depression through selecting the most appropriate level  out of five where level 1 is “no problem”, for each dimension. EQ-5D-5L VAS is self reported overall health on the day of the interview on a 0–100 hash-marked, vertical visual analogue scale. | EQ-5D-5L VAS is self reported overall health on the day of the interview on a 0–100 hash-marked, vertical visual analogue scale.  The EQ-5D-5L VAS ranges from 0 to 100 with higher VAS score indicates greater health condition. |  |  |  |  |  |
| Change from baseline on Patient Activation Measure (PAM) across 4, 8,12 and 24 weeks. | to assess the patients’ ability and willingness to take an active role in their health and healthcare. It encompasses 13 statements (e.g., “I know what each of my prescribed medications do”), each given a number of points based on the selected response. The total points across all items determine an overall score, categorised in four levels whereby level 1 is low level of activation and level 4 is defined by higher level of knowledge, skills and confidence in their care | Scoring: PAM is a 13-item survey assessing an individual's knowledge, skills, and confidence in managing their health. Responses yield a score on a 0 to 100 scale, correlating to one of four activation levels. ​  Interpretation:  Level 1: Individuals may not yet understand the importance of their role in managing their health.​  Level 2: Individuals may lack confidence and knowledge to take action.​  Level 3: Individuals are beginning to take action but may still lack confidence and skill to support new behaviours.​  Level 4: Individuals have adopted many of the behaviours but may not be able to maintain them under stress. |  |  |  |  |  |
| Change from baseline on Beck Hopelessness Scale (BHS-4) across 4, 8,12 and 24 weeks. | to measure an individual's level of hopelessness and assess patients’ cognitive outlook regarding the future. We will be using the short, four true/false statements version (e.g., “I look forward to the future with hope and enthusiasm”). Higher scores relate to more severe hopelessness | 4 item version. Each item is scored 0 or 1, consistent with the original true-or-false format. The total score ranges from 0 to 4, with higher scores indicating greater hopelessness.  Reverse-scored items: Items 1 and 4 are reverse-worded and are automatically reverse-scored within the database to ensure scoring accuracy. |  |  |  |  |  |
| Change from baseline on Readiness for Change Ruler across 4, 8,12 and 24 weeks. | in the context of motivational interviewing and behaviour change interventions. It measures an individual's readiness and willingness to make a specific change in their behaviour over three items covering importance, confidence and readiness for change on a 0-10 scale where higher scores indicate greater readiness for change | This tool assesses three subscales—readiness, confidence, and importance—each rated on a scale from 0 to 10.​  Interpretation: Higher scores indicate greater readiness to change, confidence in the ability to change, and perceived importance of the change. |  |  |  |  |  |

## **5.2 Analysis methods** _(27)_

All analyses will be conducted on an Intention-To-Treat (ITT) basis (European Medicines Agency, 1998; Kahan & Morris, 2013), i.e. all participants who are randomised whether or not they took part in any treatment or provided any follow up data, and two-tailed. Exploratory analysis will be conducted first for all measures; All participant demographic and outcome measures will be summarised by arm across follow-up times if repeatedly measured, with n (non-missing sample size), mean, standard deviation, median, inter quantile range, maximum and minimum for continuous variables, the frequency and percentages (based on the non-missing sample size) of observed levels for all categorical measures.

5.2.1 Summary of primary and secondary outcomes analysis

Treatment effect estimate and its precision (95% confidence interval) on all repeated outcomes measure will be quantified by multilevel modelling (MLM) with baseline measures and minimisation factors included as covariates, participants higher-level analytical units (European Medicines Agency, 2013; Kahan & Morris, 2013). Assumption for all linear regression will be assessed by visually and numerically exploring the residual values of each MLM modelling. No formal adjustment for multiple significance testing will be applied, in that there is only one primary outcome, and secondary outcomes will be considered supportive to the primary analysis. The significance of all parameters will be tested using 2-tailed 0.05 significance level. Skewed outcome variables will be transformed for linear regression modelling, or using quantile regression if needed, with reference on data exploratory results.

5.2.2 Analysis of primary outcome

The primary outcome is the change of PHQ-9 from baseline across 24 weeks post randomisation so the parameter (95%CI) reflecting treatment effects will be group difference of mean change from baseline across 24 weeks. Since the PHQ-9 will be repeatedly collected, the treatment effect estimate parameter(95%CI) will be derived using ANCOVA approach by means of MLM with covariate including baseline measure, minimisation factors, binary group status, follow up time, interaction term of group and time, participant will be the level 2 analytical unit, recruitment centre will be the level 3 analytical unit [6]. The proposed MLM model equation could be written as (1):

$$y_{ijk}=\beta_{0jk}+BX_{ijk}+e_{ijk}$$

$\beta_{0jk}=\beta_{0}+v_{0k}+\mu_{0jk}$ (1)

$$v_{0k}\sim N(0,\sigma_{v_{0}}^{2})$$

$$\mu_{0jk}\sim N(0,\sigma_{\mu_{0}}^{2})$$

$$e_{ijk}\sim N(0,\sigma_{e}^{2})$$

With$y_{ijk}$ is the PHQ-9 change from baseline score for $j^{th}$ participant of $k^{th}$ group at $i^{th}$ follow-up time, $i$= follow up time, $j$= participant indicator, $k$= recruitment centre indicator; X are covariates vector including baseline measure, minimisation factors , arm status, dummy coding for discrete time, and interaction terms of arm × time. B is a vector of fixed effect regression coefficients, $\beta_{0jk}$ is regression intercept parameter which was set random at participant and recruitment centre level (if any significant variability shown from model exploring), $\beta_{0}$ is the overall mean change score estimate, $v_{0k}$ is the departure of $k^{th}$ recruitment centre change score from overall mean change estimate and distributed normally with mean 0 and variance $\sigma_{v_{0}}^{2},$ $\mu_{0jk}$ is the departure of change score for $j^{th}$ participant in $k^{th}$ recruitment centre from $k^{th}$ recruitment centre mean change estimate and distributed normally with mean 0 and variance $\sigma_{\mu_{0}}^{2}$,$e_{ijk}$ is the regression residual term which follows a normal distribution with mean 0 and variance $\sigma_{e}^{2}$.

Prior to the final MLM modelling, which will yield the treatment effect estimate, a variance components model will be performed first to examine the relative variability of outcome at recruitment centre ($k$), participant ($j$) and follow-up time ($i$) level, if any higher level showed trivial variability, i.e., statistically non-significant variance at that level, or causing model convergence issues, that the level structure in the data will be removed from the above modelling (1) and the above-mentioned model will be reduced to a two-level regression model (2) or even a single level regression.

$$y_{ij}=\beta_{0j}+BX_{ij}+e_{ij}$$

$\beta_{0j}=\beta_{0}+\mu_{0j}$ (2)

$$\mu_{0j}\sim N(0,\sigma_{\mu_{0}}^{2})$$

$$e_{ij}\sim N(0,\sigma_{e}^{2})$$

In equation (2), the $i$ will be the follow-up time, $j$ will be the higher level analytical unit which has significant variance, either participant or recruitment centre. Other parameters have similar meaning as in equation (1).

For MLM, a residual Q-Q plot will be examined to check the normality assumption for linear regression modelling; for single-level regression modelling, regression diagnosis procedures will be performed to check how well the assumption for a linear regression is met in the data by means of conventional graphic and numerical approach. As participants will be recruited from multiple sites, potential site clustering will be accounted for by means of robust standard error adjusting for site clustering effects for model parameters if data exploratory analysis showed there is great variability at site level.

5.2.3 Sensitivity analyses of primary outcome

- The model shown in section 5.2.2 will be performed on observed data only and the safety dataset if the latter is different from observed dataset.
- The model shown in section 5.2.2 will be performed on imputed dataset under missing not at random assumption, with missingness imputed by controlled imputation methods such as pattern mixture approach.
- If any participant dies due to distress or the intervention (e.g. suicide), before their last follow-up assessment, the change from baseline score for those unavailable follow-ups will be replaced with 0 for a sensitivity analysis (European Medicines Agency, 2020; Kahan et al., 2024; Mészáros et al., 2024).

## 5.2.4 Secondary analysis of primary outcome

- Model shown in section 5.2.2 will be performed on per protocol population (PPA).
- Complier Average Causal Effect (CACE) estimation will be performed to explore the difference between the primary outcome in those participants who complied with the intervention and those who would have complied if assigned to treatment.

5.2.5 Planned subgroup analyses of primary outcome

There is no planned subgroup analysis.

5.2.6 Analysis of secondary outcomes

All the secondary outcomes will be analysed with same analytical modelling as for primary outcome shown in section 5.2.2.

## **5.3 Missing data**_(28)_

Missing values in all outcomes will be checked and reported across treatment group and follow up time. As the outcome will be repeatedly measured, a three level logistic regression with participants as level 2 analytical unit and recruitment centre as level 3 analytical unit will be performed to test the influence of treatment status and baseline measures on outcome missingness, with reference on result of model exploratory analysis. The missing value patterns and the results from multilevel logistic regression modelling will be used to inform missing value imputation under Missing at Random (MAR) assumption (Carpenter et al., 2023). Although multilevel modelling for repeated measures could be automatically taken into account missing outcomes under MAR assumption and may be used to give sensible results (Goldstein, 2011), to make sure all randomised participants and all missing follow-ups will be included in the analysis, the missing values will be imputed using analytical multilevel modelling to quantify the treatment effect estimates (Carpenter et al., 2011). Results of modelling on observed data will be used as sensitivity analysis to check the robustness of results sensitive to missing values (White, Horton, et al., 2011). The then latest version Stata and/or Blimp software will be used to perform multiple imputations via analytical model by means of Markov chain Monte Carlo (MCMC) approach for multilevel data (Enders et al., 2020). MCMC procedure setting include burn-in length=5000, chain length=10000, a thinning of 100 and non-informative priors for all parameters included in the model. Multiple chains will be drawn and the MCMC setting might be changed if needed after examining convergence information. A fixed seed number for MCMC run will be used at each MCMC run for results reproducibility. Twenty imputed datasets will be generated initially with possible imputing number increased after checking imputation performance (White, Royston, et al., 2011). Results from imputed dataset will be combined using Rubin’s imputation rules to produce a pooled treatment effect estimate (95% CI) and a pooled p-value for the test of null hypothesis of no treatment effect (Carpenter et al., 2023). To check the influence of missingness on treatment estimate, Missingness will be imputed by controlled imputed approach under various Missing Not Under randoms assumption.

## **5.4 Additional analyses/exploratory analysis** _(29)_

There is no other planned additional analysis.

## **5.5 Harms & Adverse events** _(30)_

The number (and percentage) of participants experiencing each AE/SAE will be presented for each treatment arm categorised by severity (across follow-up time if needed) (Ioannidis et al., 2004). For each participant, only the maximum severity experienced of each type of AE will be displayed. The number (and percentage) of occurrences of each AE/SAE will also be presented for each treatment arm. No formal statistical testing will be undertaken. The adverse event data will be additionally disaggregated by sex/gender in line with SAGER guidelines (Van Epps et al., 2022).

## **5.6 Statistical software** _(31)_

The analysis will primarily be carried out using Stata and other packages such as R or Blimp if necessary. All the software will be the then latest version available in University of Nottingham (UoN) when study data is ready for analysis. All the data will be stored in UoN secure server and analysed in UoN computers. All the data and analytic code will be archived as per instruction from study CI Dr Sam Malins who will also be the data custodians for this study.

## Supplementary Material 7: Summary of abbreviations

AE Adverse Event

CDSS Clinical Database Support Service

CI Chief Investigator

CICS Consultation Interactions Coding Scheme

CRF Case Report Form

CSRI Client Service Receipt Inventory

DMC Data Monitoring Committee

EMCA CPH East Midlands Cancer Alliance Centre for Psychological Health

GAD-7 Generalised Anxiety Disorder – 7 items

GCP Good Clinical Practice

MAR Missing At Random

MCID Minimally Clinically Important Difference

NHS R&D National Health Service Research & Development

NICE National Institute for Health and Care Excellence

PHQ-9 Patient Health Questionnaire – 9 items

PIS Participant Information Sheet

PPI/E Patient and Public Involvement and Engagement

RDN Research Delivery Network

SAE Serious Adverse Event

SAP Statistical Analysis Plan

SWEMWBS Short Warwick-Edinburgh Mental Wellbeing Scale

TAU Treatment As Usual

TMF Trial Master File

TMG Trial Management Group

TPI Therapy Preparation Intervention

TPM Therapy Preparation Messages

TPS Therapy Preparation Session

TSC Trial Steering Committee

WSAS Work and Social Adjustment Scale

## References

Adamson, J., Ali, S., Santhouse, A., et al. (2020). Cognitive behavioural therapy for chronic fatigue and chronic fatigue syndrome: Outcomes from a specialist clinic in the UK. Journal of the Royal Society of Medicine, 113(10), 394–402.

Andreescu, C., Lenze, E. J., Dew, M. A., et al. (2007). Effect of comorbid anxiety on treatment response and relapse risk in late life depression: Controlled study. British Journal of Psychiatry, 190(4), 344–349.

Beecham, J., & Knapp, M. (2001). Costing psychiatric interventions. In G. Thornicroft (Ed.), Measuring mental health needs. Gaskell.

Cameron, C. L. (2007). Single session and walk in psychotherapy: A descriptive account of the literature. Counselling and Psychotherapy Research, 7(4), 245–249.

Carpenter, J. R., Bartlett, J. W., Morris, T. P., et al. (2023). Multiple imputation and its application (2nd ed.). John Wiley & Sons.

Carpenter, J. R., Goldstein, H., & Kenward, M. G. (2011). REALCOM IMPUTE software for multilevel multiple imputation with mixed response types. (Software).

Carter, O., Pannekoek, L., Fursland, A., et al. (2012). Increased wait list time predicts dropout from outpatient enhanced cognitive behaviour therapy (CBT E) for eating disorders. Behaviour Research and Therapy, 50(7), 487–492.

DiMatteo, M. R., & Haskard Zolnierek, K. B. (2010). Impact of depression on treatment adherence and survival from cancer. In Depression and cancer (pp. 101–124). John Wiley & Sons. https://onlinelibrary.wiley.com/doi/abs/10.1002/9780470972533.ch5

Enders, C. K., Du, H., & Keller, B. T. (2020). A model based imputation procedure for multilevel regression models with random coefficients, interaction effects, and nonlinear terms. Psychological Methods, 25(1), 88–105.

European Medicines Agency. (1998). Statistical principles for clinical trials (E9): ICH tripartite guideline. European Medicines Agency.

European Medicines Agency. (2013). Guideline on adjustment for baseline covariates. European Medicines Agency.

European Medicines Agency. (2016). Guideline on multiplicity issues in clinical trials. European Medicines Agency.

European Medicines Agency. (2020). ICH E9 (R1) addendum on estimands and sensitivity analysis in clinical trials to the guideline on statistical principles for clinical trials. European Medicines Agency.

Forbes, H., Carreira, H., Funston, G., et al. (2024). Early, medium and long term mental health in cancer survivors compared with cancer free comparators: Matched cohort study using linked UK electronic health records. eClinicalMedicine, 76, 102826.

Furukawa, T. A., Noma, H., Caldwell, D. M., et al. (2014). Waiting list may be a nocebo condition in psychotherapy trials: A contribution from network meta analysis. Acta Psychiatrica Scandinavica, 130(3), 181–192.

Gamble, C., Krishan, A., Stocken, D., et al. (2017). Guidelines for the content of statistical analysis plans in clinical trials. JAMA, 318(23), 2337–2343.

Gerhards, S. A., Huibers, M. J., Theunissen, K. A., de Graaf, L. E., Widdershoven, G. A., & Evers, S. M. (2011). The responsiveness of quality of life utilities to change in depression: A comparison of instruments (SF 6D, EQ 5D, and DFD). Value in Health, 14(5), 732–739.

Goldstein, H. (2011). Multilevel statistical models (4th ed.). John Wiley & Sons.

Ioannidis, J. P. A., Evans, S. J. W., Gøtzsche, P. C., et al. (2004). Better reporting of harms in randomized trials: An extension of the CONSORT statement. Annals of Internal Medicine, 141(10), 781–788.

Kahan, B. C., Hindley, J., Edwards, M., et al. (2024). The estimands framework: A primer on the ICH E9 (R1) addendum. BMJ, 384.

Kahan, B. C., & Morris, T. P. (2013). Analysis of multicentre trials with continuous outcomes: When and how should we account for centre effects? Statistics in Medicine, 32(7), 1136–1149.

Malins, S., Moghaddam, N., Morriss, R., et al. (2020). Patient activation in psychotherapy interactions: Developing and validating the consultation interactions coding scheme. Journal of Clinical Psychology, 76(4), 646–658.

Mander, J., Wittorf, A., Teufel, M., Schlarb, A., Hautzinger, M., Zipfel, S., et al. (2012). Patients with depression, somatoform disorders, and eating disorders on the stages of change: Validation of a short version of the URICA. Psychotherapy, 49(4), 519–527.

McGrath, R. E. (2019). The VIA Assessment Suite for Adults: Development and initial evaluation (Revised ed.). VIA Institute on Character. https://www.viacharacter.org/pdf/Technical%20Report%20Revised%20Edition%202019_1.pdf

Mészáros, L., Lasch, F., Delafont, B., et al. (2024). Estimands in CNS trials: A review of strategies for addressing intercurrent events. Contemporary Clinical Trials Communications, 38, 101266.

Miller, S. D., Chow, D. E., Malins, S. E., & Hubble, M. A. (2023). The field guide to better results: Evidence based exercises to improve therapeutic effectiveness. American Psychological Association.

Miller, W. R., & Rollnick, S. (2002). Motivational interviewing: Preparing people for change (2nd ed.). Guilford Press.

Morriss, R., Garland, A., Nixon, N., et al. (2016). Efficacy and cost effectiveness of a specialist depression service versus usual specialist mental health care to manage persistent depression: A randomised controlled trial. The Lancet Psychiatry, 3(9), 821–831.

NHS Digital. (2024). NHS Talking Therapies, for anxiety and depression: Annual reports, 2022 to 23. https://digital.nhs.uk/data-and-information/publications/statistical/nhs-talking-therapies-for-anxiety-and-depression-annual-reports/2022-23

National Institute for Health and Care Excellence. (2019). Position statement on use of the EQ 5D 5L value set for England (updated October 2019). https://www.nice.org.uk/about/what-we-do/our-programmes/nice-guidance/technology-appraisal-guidance/eq-5d-5l

PSSRU. (2019). Unit costs of health and social care 2019. https://www.pssru.ac.uk/project-pages/unit-costs/unit-costs-2019/

Pitman, A., Suleman, S., Hyde, N., et al. (2018). Depression and anxiety in patients with cancer. BMJ, k1415.

Ramsey, S. D., Willke, R. J., Glick, H., Reed, S. D., Augustovski, F., Jonsson, B., et al. (2015). Cost effectiveness analysis alongside clinical trials II: An ISPOR Good Research Practices Task Force report. Value in Health, 18(2), 161–172.

Reitzel, L. R., Stellrecht, N. E., Gordon, K. H., et al. (2006). Does time between application and case assignment predict therapy attendance or premature termination in outpatients? Psychological Services, 3(1), 51–60.

Schleider, J. L., Zapata, J. P., Rapoport, A., et al. (2025). Single session interventions for mental health problems and service engagement: Umbrella review of systematic reviews and meta analyses. Annual Review of Clinical Psychology. Advance online publication. https://doi.org/10.1146/annurev-clinpsy-081423-025033

Singla, D. R., Schleider, J. L., & Patel, V. (2023). Democratizing access to psychological therapies: Innovations and the role of psychologists. Journal of Consulting and Clinical Psychology, 91(11), 623–625.

Steinert, C., Stadter, K., Stark, R., et al. (2017). The effects of waiting for treatment: A meta analysis of waitlist control groups in randomized controlled trials for social anxiety disorder. Clinical Psychology and Psychotherapy, 24(3), 649–660.

Suh, J. W., Bell, V., Buckman, J. E., et al. (2025). A record linkage study of post stroke primary care psychological therapy effectiveness in England. Nature Mental Health, 1–10.

Van Epps, H., Astudillo, O., Martin, Y. D. P., et al. (2022). The sex and gender equity in research (SAGER) guidelines: Implementation and checklist development. European Science Editing, 48, e86910. https://ese.arphahub.com/article_preview.php?id=86910

Vierron, E., & Giraudeau, B. (2019). Design effect in multicenter studies: Gain or loss of power? BMC Medical Research Methodology. https://link.springer.com/article/10.1186/1471-2288-9-39

Wells, C., Malins, S., Clarke, S., et al. (2020). Using smart messaging to enhance mindfulness based cognitive therapy for cancer patients: A mixed methods proof of concept evaluation. Psycho Oncology, 29(1), 212–219.

White, I. R., Horton, N. J., Carpenter, J., et al. (2011). Strategy for intention to treat analysis in randomised trials with missing outcome data. BMJ, 342.

White, I. R., Royston, P., & Wood, A. M. (2011). Multiple imputation using chained equations: Issues and guidance for practice. Statistics in Medicine, 30(4), 377–399.
