# Supplementary material for: Preparation for online psychological therapy for depression in people living with and beyond cancer in East Midlands NHS primary and secondary care services in England: protocol for the PROSPER randomised controlled trial
Source: BMJ Open. 2026 May 18;16(5):e108442. doi: 10.1136/bmjopen-2025-108442 (PMC13185027; doi:10.1136/bmjopen-2025-108442)
Supplement: Supplementary file 2 [file bmjopen-2025-108442.reviewer_comments.pdf]

PEER REVIEW HISTORY

BMJ Open publishes all reviews undertaken for accepted manuscripts. Reviewers are asked to complete a checklist review form and are provided with free text boxes to elaborate on their assessment. These free text comments are reproduced below.

ARTICLE DETAILS

Title (Provisional)

Preparation for online psychological therapy for depression in people living with and beyond cancer in East Midlands NHS primary and secondary care services in England: protocol for the PROSPER randomised controlled trial

Authors

Boutry, Clement Olivier; Hagyari-Donaldson, Paulina; Hill, Adam; Mauger, Fallon; Mays, Chloe; Macauley, Catherine; Covington, Melissa; Wynn, Rebecca; Simpson, Karen; Cordrey, Melanie; Higton, Fred; Hall, Charlotte L; Rathbone, James; Guo, Boliang; Patel, Shireen; Malins, Sam; Moghaddam, Nima; Morriss, Richard

---

VERSION 1 - REVIEW

**Reviewer**	**1**
**Name**	**Tachimori, Hisateru**
**Affiliation**	**Keio University School of Medicine, Tokyo, Japan**
**Date**	**24-Jan-2026**
**COI**	**None**

Overall Assessment

This study addresses an important clinical problem, mental-health deterioration and treatment dropout among cancer patients who must wait for psychological therapy, by evaluating a novel approach that combines a single-session preparation intervention (Therapy Preparation Intervention, TPI) with smart-messaging. I consider this to be a highly worthwhile contribution.

The pragmatic, multi-centre randomised controlled trial design enhances external validity (generalizability). Selecting the Patient Health Questionnaire-9 (PHQ-9) as the primary outcome and planning repeated assessments over 24 weeks, analysed with multilevel modelling, is appropriate. The mixed-methods component that incorporates quantitative measures of patient activation, readiness for change, and hope together with qualitative interviews is likely to shed valuable light on the mechanisms underlying any observed effects.

The protocol is generally well written; however, a few relatively minor points would benefit from additional clarification:

1. Design effect in the sample-size calculation

Please provide a brief justification for the design-effect factor that was applied (e.g., the assumed intra-class correlation and the degree of unequal recruitment across sites). A short description of how this value was derived will help readers assess the adequacy of the planned sample size.

2. Handling of missing data

It is commendable that the authors have pre-specified the use of multiple imputation (MI) for handling missing observations. While detailed MI specifications need not appear in the protocol itself, they should be documented in the forthcoming Statistical Analysis Plan (SAP). Stating explicitly that the SAP—finalised before any data are released—will contain a full description of the MI method (imputation model, number of imputations, variables included, etc.) would strengthen methodological transparency.

3. Sensitivity-analysis plan

The protocol should include a more concrete outline of the planned sensitivity analyses (e.g., alternative missing-data assumptions, different correlation structures in the mixed model, per-protocol versus intention-to-treat analyses). Providing at least a high-level description will reassure reviewers that the robustness of the primary findings will be systematically examined.

4. Definition of “cancer patient” within the eligibility criteria

The inclusion criterion simply states “individuals living with or beyond cancer.” Please clarify how the authors define “cancer patient” for this trial:

Are all tumour sites and histological types eligible, or are specific cancers excluded? Is disease stage (e.g., early-stage vs. metastatic) taken into account? Does current treatment status (receiving chemotherapy, radiotherapy, immunotherapy, hormonal therapy, or being in surveillance) affect eligibility?

A concise description of these parameters will improve the reproducibility of the recruitment process and help readers understand to which patient population the results can be generalised.

Addressing these points will further improve the clarity and rigor of the protocol without detracting from its overall strengths.

**Reviewer**	**2**
**Name**	**Jin, Jingfen**

Affiliation **Zhejiang University School of Medicine Second Affiliated Hospital, Nursing Department**

Date **24-Jan-2026**

COI **None**

Dear Editors,

Thank you for the invitation to review this protocol manuscript. I have evaluated the paper and appreciate the opportunity to provide feedback. Unfortunately, I recommend rejection in its current form, primarily due to concerns regarding clarity, methodological rigor, and presentation, which collectively affect the overall strength and readability of the manuscript.

1.Title: The study focuses on cancer patients with depression, yet this specific population is not clearly reflected in the title. A more precise title would better convey the scope of the research.

2.Keywords: The current list contains seven keywords, some of which are redundant (e.g., “cancer” and “oncology”). Streamlining these and including the term “protocol” could improve reader orientation.

3.Background section: (1) The concept of “brief preparatory interventions” is introduced without sufficient detail regarding their core components, delivery formats, or proposed mechanisms. (2) The justification for selecting motivational interviewing (MI) as the intervention requires further development. It would be helpful to explain why MI is particularly suitable for enhancing therapy readiness and engagement in cancer patients with depression, and how it aligns with the study’s objectives. (3) The background would benefit from a more thorough discussion of prior evidence supporting the use of MI in similar contexts—for instance, clinical trials or meta-analyses demonstrating its efficacy among cancer patients awaiting psychological therapy, especially in comparison to other preparatory approaches.

4.Participant eligibility: Additional details regarding the psychiatric history of participants would be valuable—for example, whether individuals with other mental health conditions are included or excluded. It would also be helpful to know whether participants are concurrently undergoing cancer treatments (e.g., chemotherapy, radiation) during the waiting period for psychological therapy. Furthermore, the use of stratified randomization to balance potential confounding factors between groups should be considered and described.

5.Please provide an updated SPIRIT checklist (2025 version) to ensure adherence to current reporting standards for trial protocols.

6.Ethical considerations: The control group is described as receiving no reminders, assessments, or follow-ups during the waiting period. This raises a potential ethical concern regarding the risk of worsening depression in this group.Additionally, the regular messaging planned for the intervention group may themselves exert a supportive effect. To better

isolate the effect of MI, consider providing the control group with similar non-therapeutic communications to control for attention and contact effects.

VERSION 1 - AUTHOR RESPONSE

Reviewer: 1
Hisateru Tachimori, Keio University School of Medicine, Tokyo, Japan

Note: For Q1-3 – we have added the SAP in the supplementary material. We refer directly to the SAP to answer the queries.

1. Design effect in the sample-size calculation
Please provide a brief justification for the design-effect factor that was applied (e.g., the assumed intra-class correlation and the degree of unequal recruitment across sites). A short description of how this value was derived will help readers assess the adequacy of the planned sample size.

Please see SAP section 2.3 for the details of sample size calculation.

2. Handling of missing data
It is commendable that the authors have pre-specified the use of multiple imputation (MI) for handling missing observations. While detailed MI specifications need not appear in the protocol itself, they should be documented in the forthcoming Statistical Analysis Plan (SAP). Stating explicitly that the SAP—finalised before any data are released—will contain a full description of the MI method (imputation model, number of imputations, variables included, etc.) would strengthen methodological transparency.

Please see SAP section 5.3 about details of imputation procedure.

3. Sensitivity-analysis plan
The protocol should include a more concrete outline of the planned sensitivity analyses (e.g., alternative missing-data assumptions, different correlation structures in the mixed model, per-protocol versus intention-to-treat analyses). Providing at least a high-level description will reassure reviewers that the robustness of the primary findings will be systematically examined.

Please see SAP section 5.2.3 and 5.2.4 about sensitivity analysis.

4. Definition of “cancer patient” within the eligibility criteria
The inclusion criterion simply states “individuals living with or beyond cancer.” Please clarify how the authors define “cancer patient” for this trial:
Are all tumour sites and histological types eligible, or are specific cancers excluded? Is disease stage (e.g., early-stage vs. metastatic) taken into account? Does current treatment status (receiving chemotherapy, radiotherapy, immunotherapy, hormonal therapy, or being in surveillance) affect eligibility?

A concise description of these parameters will improve the reproducibility of the recruitment process and help readers understand to which patient population the results can be generalised.

We have clarified the definition of "cancer patient" in the eligibility criteria to improve reproducibility and interpretability. In this trial, eligibility includes adults with a diagnosis of cancer (solid or haematological), past or present, recruited via NHS cancer care/psychological therapy referral pathways. We have specified that tumour site, histological type, disease stage (including metastatic disease), and treatment status (active treatment, maintenance therapy, or surveillance) do not restrict eligibility.

These characteristics are not used as screening criteria and are not collected as baseline trial variables; this has been clarified in the manuscript and noted in the Strengths and limitations box.

Reviewer: 2
Dr. Jingfen Jin, Zhejiang University School of Medicine Second Affiliated Hospital

1.Title: The study focuses on cancer patients with depression, yet this specific population is not clearly reflected in the title. A more precise title would better convey the scope of the research.

We agree that the original title did not sufficiently reflect the specific target population. We have revised the title to explicitly indicate that the trial focuses on depression in people living with and beyond cancer: Preparation for online psychological therapy for depression in people living with and beyond cancer: the PROSPER randomised controlled trial protocol.

2.Keywords: The current list contains seven keywords, some of which are redundant (e.g., "cancer" and "oncology"). Streamlining these and including the term "protocol" could improve reader orientation.

We agree that the previous keywords included redundancy (e.g., "cancer" and "oncology"). We have streamlined the list accordingly and added "protocol" to clearly indicate the article type and improve indexing. The revised keywords are: Online psychological therapy; Depression; Cancer; Protocol; Motivational interviewing; Cognitive behavioural therapy; Randomised controlled trial.

3.Background section: (1) The concept of "brief preparatory interventions" is introduced without sufficient detail regarding their core components, delivery formats, or proposed mechanisms. (2) The justification for selecting motivational interviewing (MI) as the intervention requires further development. It would be helpful to explain why MI is particularly suitable for enhancing therapy readiness and engagement in cancer patients with depression, and how it aligns with the study's objectives. (3) The background would benefit from a more thorough discussion of

prior evidence supporting the use of MI in similar contexts—for instance, clinical trials or meta-analyses demonstrating its efficacy among cancer patients awaiting psychological therapy, especially in comparison to other preparatory approaches.

We have strengthened the Background in three ways.
(1) Brief preparatory interventions: *We now describe their typical components, delivery formats, and proposed mechanisms (e.g., expectation setting, aligning expectations, goal clarification, and troubleshooting barriers to engagement), and summarise wait-list evidence indicating that brief inputs during waiting periods may initiate change and mitigate deterioration.*
(2) Rationale for MI: *We expanded the rationale for selecting MI by linking its focus on ambivalence, motivation, and commitment to the challenges of engagement when depressive symptoms are present, and to our prespecified mechanistic outcomes (hope, readiness for change, and patient activation).*
(3) Prior evidence base: *We provide a fuller account of prior evidence relevant to MI-based preparation, including MI used as a pretreatment integrated with CBT, evidence supporting MI as a brief/single-session approach, and evidence of MI reducing dropout in cancer/chronic pain psychological interventions. We also clarify that the effectiveness of MI-based preparation specifically during waiting periods for psychological therapy among people affected by cancer has not been well established, motivating the present trial.*

4.Participant eligibility: Additional details regarding the psychiatric history of participants would be valuable—for example, whether individuals with other mental health conditions are included or excluded. It would also be helpful to know whether participants are concurrently undergoing cancer treatments (e.g., chemotherapy, radiation) during the waiting period for psychological therapy. Furthermore, the use of stratified randomization to balance potential confounding factors between groups should be considered and described.

We have clarified participant eligibility regarding psychiatric comorbidity and cancer treatment status. The trial uses pragmatic eligibility criteria: comorbid mental health conditions are not excluded unless they raise immediate safety concerns or indicate that the individual is not clinically appropriate for the host pathway; this is assessed during screening by the referring clinical team, with "immediate risk to self or others" as an exclusion criterion. Participants may also be receiving active cancer treatment or be in surveillance during the waiting period; treatment status is not used to restrict eligibility.

Regarding allocation, we do not use traditional stratified randomisation; instead, we use minimisation to balance key potential confounders between arms. Specifically, the secure web-based randomisation system applies minimisation by study site and baseline GAD-7 clinical cut-off (≥8), which reduces the risk of imbalance without creating sparse strata.

5.Please provide an updated SPIRIT checklist (2025 version) to ensure adherence to current reporting standards for trial protocols.

Thank you. We have updated the protocol reporting checklist in line with the SPIRIT 2025 statement and have provided the completed SPIRIT 2025 checklist as a supplementary file to ensure adherence to current reporting standards. We also reviewed the protocol against SPIRIT

2025's updated emphasis on open science and patient/public involvement and confirmed these elements are addressed in the protocol text and/or supplementary materials.

6.Ethical considerations: The control group is described as receiving no reminders, assessments, or follow-ups during the waiting period. This raises a potential ethical concern regarding the risk of worsening depression in this group. Additionally, the regular messaging planned for the intervention group may themselves exert a supportive effect. To better isolate the effect of MI, consider providing the control group with similar non-therapeutic communications to control for attention and contact effects.

Thank you for raising these ethical and methodological considerations. We agree that prolonged waiting can be associated with symptom worsening, which is one reason this trial is needed. Importantly, the control arm is treatment as usual (TAU) within existing NHS pathways rather than "no contact". All participants (both arms) complete the same research assessments at 4, 8, 12, and 24 weeks post-randomisation, with automated REDCap reminders for questionnaire completion, and participants may report adverse events during assessments or via clinicians.

In addition, all participants receive the host service's written guide at the start of the waiting period outlining what to expect from therapy and practical preparation advice.

Participant safety is supported through routine clinical screening by the referring team (including exclusion of those at immediate risk to self or others), adverse event monitoring, and standard NHS escalation pathways as needed.

We acknowledge that the intervention's messaging component could have non-specific supportive effects. However, the intervention is intentionally designed as a package (MI-informed preparation session plus personalised smart-messaging) to enhance readiness and engagement during the waiting period. Therefore, the primary comparison is against TAU to maximise real-world applicability and estimate the incremental benefit of implementing this intervention package within routine care. We have clarified TAU contacts (in the Methods section within the TAU description) and safety procedures (under Safety outcome measures) in the manuscript and acknowledge the possibility of non-specific effects of messaging when interpreting outcomes (in the strengths and limitations).
